# Impact of the COVID-19 pandemic on small vulnerable newborns: an interrupted time series analysis in Peru and Brazil

**DOI:** 10.7189/jogh.15.04026

**Published:** 2025-01-31

**Authors:** Kim N Cajachagua-Torres, Mariana Otero Xavier, Hugo G Quezada-Pinedo, Carlos A Huayanay-Espinoza, Alvaro Gonzalo Oviedo Rios, Agbessi Amouzou, Abdoulaye Maïga, Nadia Akseer, Alicia Matijasevich, Luis Huicho

**Affiliations:** 1 Department of Pediatrics, New York University Grossman School of Medicine, New York, New York, USA; 2 Department of Pediatrics, Erasmus MC, University Medical Centre Rotterdam, Rotterdam, the Netherlands; 3 Centro de Investigación en Salud Materna e Infantil and Centro de Investigación para el Desarrollo Integral y Sostenible, Universidad Peruana Cayetano Heredia, Lima, Peru; 4 Departamento de Medicina Preventiva, Faculdade de Medicina FMUSP, Universidade de São Paulo, São Paulo, Brazil; 5 Department of Population Health Sciences, Duke University School of Medicine, Durham, North Carolina, USA; 6 Department of International Health, Johns Hopkins University Bloomberg School of Public Health, Baltimore, Maryland, USA; 7 Facultad de Medicina, Universidad Peruana Cayetano Heredia, Lima, Peru

## Abstract

**Background:**

We examined COVID-19’s impact on the number of small vulnerable newborns (SVN) at national and regional levels in Peru and Brazil.

**Methods:**

Using national birth registries, we examined monthly numbers of preterm (PT), low birthweight (LBW), and small for gestational age (SGA) newborns. We analysed COVID-19’s impact on SVN using two interrupted time series models. We estimated SVN’s expected numbers in the absence of the pandemic using mixed-effects regressions and calculated percent changes by comparing these estimates to observed during the pandemic. Incidence rate ratios (IRR) were estimated using Poisson regression.

**Results:**

In Peru, the average percent changes in PT, LBW, and SGA births were −17%, −11%, and −3% in 2020, and −10%, −4%, and +2% in 2021, respectively. The IRR of PT and LBW declined throughout the pandemic, while SGA IRR increased in August 2020–November 2020 and May 2021–December 2021. The Coast region experienced the greatest drop in PT, LBW, and SGA IRR in 2020, followed by a slight increase in 2021, whereas the Highlands and Amazon regions had increased LBW and SGA IRR. In Brazil, the percent changes in PT, LBW, and SGA births were +1%, −3%, and −8% in 2020, and +1%, 0%, and −1% in 2021, respectively. Most PT, LBW, and SGA IRRs decreased during the pandemic, except in the Northeast and Southeast regions, where PT increased in 2020. All regions experienced declines in LBW and SGA in 2020, with the Central-West and South regions showing the greatest LBW declines and Central-West region the highest SGA decrease.

**Conclusions:**

No significant worsening of neonatal outcomes were observed during the COVID-19 pandemic. In Peru, PT and LBW births declined, while SGA increased from August 2020. In Brazil, PT births slightly increased, while LBW and SGA births declined in 2020, remaining stable in 2021.

In 2020, 19.8 million low birthweight (LBW), 13.4 million preterm (PT), and 23.4 million small for gestational age (SGA) babies were born worldwide [1]. These so-called small vulnerable newborns (SVN) have a high risk of mortality and of long-term health consequences [1,2]. In 2020, the SVN phenotypes were responsible for 1.3 million neonatal deaths globally [1,3]. Among these deaths, 73% were attributed to PT births, while the remaining were attributed to SGA births [1,4–9] LBW can be caused by short pregnancy gestation (<37 weeks of gestation) resulting in a PT birth, or by foetal growth restriction leading to a SGA birth [3]. The global nutrition target of reducing by 30% the prevalence of LBW is currently not on track [1], due to inadequate implementation of preventive interventions and to health system-related factors such as health workforce, health care insurance, and accountability bottlenecks [1].

In South-America, Peru has shown an average prevalence of 6.2, 5.4, and 6.6% for LBW, SGA, and PT newborns, respectively, across the period 2015–2020 [4,10]. Brazil is among the 13 countries that witnessed a decline of 0.5% or more in the average annual rate of PT birth reduction between 2010 and 2020. Despite this positive trend, its prevalence remained high at around 11% during this period, making it one of the nations with the highest rates of prematurity globally [11]. The prevalence of LBW increased from 7.8 in 2000 to 8.4% in 2015 in Brazil [12]. There is scarcity of data on the temporal evolution of the SGA phenotype in Brazil, but there is evidence of an average prevalence of 9.2% of SGA over the period 2011–2018 [13].

The COVID-19 pandemic might have influenced birth outcomes in different ways. Peru and Brazil experienced some of the highest excess mortality rates in Latin America and globally, highlighting the need for studies evaluating the influence of the COVID-19 pandemic on the SVN in these countries [14–16]. According to earlier reports, the COVID-19 pandemic had an enormous direct and indirect impact on maternal and child health [16]. Containment measures adopted during the COVID-19 pandemic might have disproportionally affected maternal and neonatal health, especially in low- and middle-income countries (LMICs) [4,17,18]. While Peru confirmed its first COVID-19 case on 6 March 2020, and implemented its containment measures on 15 March 2020 [16], Brazil implemented them on 3 February 2020, with the first confirmed COVID-19 case reported on 25 February 2020 [19]. Peru and Brazil faced significant health challenges during the COVID-19 pandemic for several reasons, including preexisting structural limitations in their health care systems, socioeconomic conditions, political responses, and public health infrastructure [4,20–22]. Both countries, in addition to confronting severe COVID-19 pandemic scenarios – Peru with the highest cumulative mortality rate and Brazil with the second highest absolute number of deaths – have also experienced significant political instability in recent years [16,23]. Previous studies in Peru and Brazil have reported that the COVID-19 pandemic was associated with reduced antenatal care visits and related procedures and increased maternal mortality [22,24,25]. On the other hand, the COVID-19 disease has been associated with increased risk of stillbirths [4]. Other authors suggest that measures such as COVID-19-related lockdowns might reduce the risk of LBW, PT and SGA births by decreasing air pollution and by lowering exposure to non-COVID infections [4,26]. However, there is a scarcity of studies examining the influence of the COVID-19 pandemic on the frequency of SVN phenotypes in LMICs.

Thus, this study aimed to assess the impact of the COVID-19 pandemic on the national and regional trends and the magnitude of changes in SVN in Peru and Brazil from 2017 to 2021. The results might provide relevant information regarding the magnitude of the direct and indirect impact of COVID-19 on LBW, PT and SGA phenotypes. Additionally, they can inform decision-makers and health workers about areas at higher risk of influence and help prepare for future events.

## METHODS

### Study design and study settings

**Figure 1:**
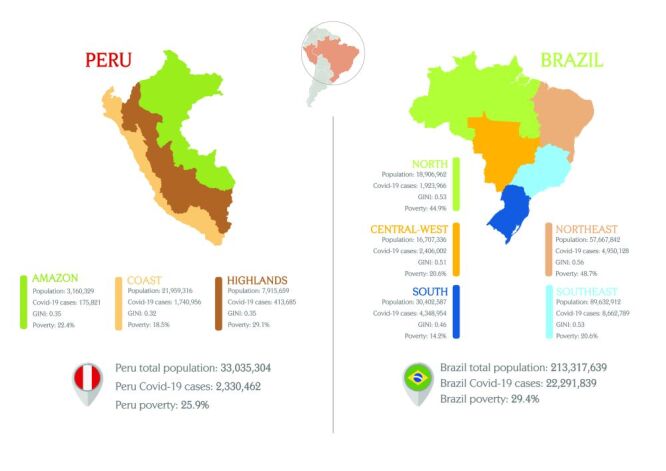
Global characteristics of Peru and Brazil with regional division. Reports the estimated total population in 2021 and the total number of covid-19 cases in 2020 and 2021.

This study used interrupted time series analysis, a quasi-experimental design, to evaluate the impact of the COVID-19 pandemic over a time by analysing data collected at regular intervals before and after the event (i.e. COVID-19 pandemic) on SVN outcomes in Peru and Brazil. By comparing both periods, this method may suggest that the event had a statistically significant impact on the outcome of interest. Peru is an upper-middle income country in South America with an estimated population of 33 035 304 inhabitants, with a gross domestic product (GDP) per capita of 6635.5 USD in 2021 [27,28]. Peru is geographically divided in three natural regions (Coast, Highlands, and Amazon). The Coast is extended along the Pacific Ocean, the Highlands are extended surrounding the Andes, and the Amazon is extended around the Amazon rainforest. In 2021, 25.9% of the population was living in poverty; with a Gini index of household income per person of 0.438 [29]. Peru has substantial remaining social inequalities in maternal and child health, and has also been one of the countries most affected by the COVID-19 pandemic worldwide [27,30]. Up to December 2021, Peru had reported 2 330 462 COVID-19 cases and 202 690 deaths [31] (**Figure 1**).

Brazil is the largest country in South America and the fifth most populous in the world, with an estimated population of 213 million inhabitants, it has a GDP of 1649.62 USD billion in 2021 (GDP per capita of 8621.73 USD) [32]. The country is divided into five geographic regions (North, Northeast, Central-West, Southeast, and South) where 27 federation units are distributed. Brazil has a large social inequality, with approximately 29.4% of the population living under 5.5 USD purchasing power parity (PPP) in 2021, and a Gini index of household income per person of 0.544 in 2021 [33]. By December 2021, 22 291 839 COVID-19 cases and 619 334 deaths from COVID-19 had been reported in Brazil [34] (**Figure 1**).

### Data source

Data were obtained from the national health information systems of Peru and Brazil. From both data sources, we used sex at birth, birthweight, and gestational weeks at birth.

The Peruvian data was extracted from the Peruvian Online Livebirth Certificate Registration System (Sistema de Registro de Nacido de Vivo in Spanish). This system includes individual information on maternal and newborn characteristics recorded immediately after delivery across all the Peruvian territory, and by all health care public and private providers [35,36]. It does not include information on stillbirths. The coverage information consistently remained above 81% across the study period [37]. We retrieved data on reported livebirths between 1 January 2017 and 31 December 2021.

The Brazilian data was extracted from the Brazilian Information System on Livebirths (Sistema de Informações sobre Nascidos Vivos, SINASC, in Portuguese) by using the National Health System Information Technology Department (DATASUS) of the Ministry of Health of Brazil [38]. We extracted information on pregnancies and newborns at federation unit level. The system captures information from the Declaration of Live Birth, a legal document filled out by the health care professional present during delivery. Encompassing over 98% of the Brazilian territory, the SINASC boasts high and homogeneous coverage, extending to over 90% of live births in most federation units [39].

### Operational definitions and exclusion criteria

The SVN outcomes considered in this study were PT, LBW and SGA. A PT birth was defined as the number of babies born alive before 37 weeks of pregnancy are completed. LBW newborns were defined as birthweight below 2500 g. A SGA newborn was defined using the INTERGROWTH-21st international newborn size for age and sex standards, which was extended to include newborns from 22 to 44 weeks gestational age, as described elsewhere [3,40]. We excluded records with missing information on gestational age at birth, birthweight, or newborn sex. We further excluded implausible birthweights defined as <250 g or ≥6500 g as they are incompatible with life or are likely due to typographical errors. Taking into account all the exclusion criteria mentioned above, in Peru, 3 856 933 livebirths were registered in the Peruvian Online Livebirth Certificate Registration System from 1 January 2017 to 31 December 2021. Of them, 1082 were excluded, resulting in 3 855 041 livebirths for analysis. In Brazil, 14 124 859 livebirths were registered in SINASC from 1 January 2017 to 31 December 2021. Of these, 228 932 were excluded, resulting in 13 895 927 livebirths for analysis.

### Statistical analysis

We performed descriptive analysis by assessing annual, quarterly, and monthly absolute numbers of PT, LBW and SGA livebirths from January 2017 to December 2021. Then, we implemented two distinct approaches of interrupted time series (ITS) to assess the COVID-19 impact on PT, LBW, and SGA from March 2020 to December 2021 in Peru and Brazil. We estimated the expected numbers of each outcome in the absence of pandemic by using mixed-effects linear regression. Subsequently, we compared the pre-pandemic (January 2017–February 2020) observed numbers to those that would be expected in the absence of the pandemic (March 2020 to December 2021) and calculated the percent changes. Finally, we estimated IRR by using Poisson regression. The ITS analysis is particularly valuable for assessing the effectiveness of population-level events at a clearly defined time point. This approach is especially useful when a 'natural experiment' occurs in a real-world context [41], such as during the COVID-19 pandemic. Similar to previous studies, we selected 60 months with March 2020 as the point of interruption. We therefore considered a three-year period for the pre-pandemic phase and a three-year period for the post-pandemic phase [18,42].

First, we used mixed-effects ordinary least squares regression models to estimate the expected numbers of SVN outcomes in the absence of pandemic. These regressions were conducted separately for each country, using the total monthly numbers of each type of SVN phenotype (PT, LBW, and SGA). We fitted a model covering the entire period to establish temporal trends. Subsequently, we applied the same model to the pre-COVID-19 period (January 2017 to February 2020). Utilising this second model, we forecasted the predicted (expected) number of each outcome (PT, LBW, and SGA livebirths) during the COVID-19 period in the hypothetical absence of COVID-19. We fitted these regressions using the monthly number of each type SVN phenotype at national and regional level as the dependent variable. This model included random intercept and slope for time trends, for accounting multiple measurements and incorporating a time variable defined in months (from January 2017 to December 2021) to capture time trends. Additionally, based on previous literature, we adjusted for each calendar month – as a dummy variable – to control for seasonality, number of livebirths to ensure that observed changes were related to the pandemic rather than population dynamics, administrative level, and COVID-19-month from March 2020 to December 2021 [18,43,44]. Then, we computed the percent change between the observed and predicted (or expected) values during the pandemic at national and regional level using the equation: (100 * (Observed value – Predicted value)/Predicted value).

Second, we estimated the monthly IRR of PT, LBW, and SGA newborns during the COVID-19 pandemic period using Poisson Generalized Linear Models with a log-link function, and a scaling adjustment to correct for overdispersion.[45] These regressions were conducted separately for each country, using the monthly total numbers of each type of SVN phenotype (PT, LBW, and SGA). The data was modelled across two distinct time periods: the ‘pre-event period’ (pre-pandemic, January 2017 to February 2020), and the ‘event period’ (encompassed the COVID-19 pandemic, from March 2020 to December 2021). To account for long-term trends and potential seasonality, we included a linear time trend and Fourier terms into the estimation model [45]. To address heteroscedasticity and autocorrelation, we used Heteroscedasticity and Autocorrelation Consistent errors (HAC). The HAC method effectively adjusts the standard errors and statistical tests by estimating weighting matrices [46].

As a supplementary analysis to examine the robustness of our findings, we adjusted additionally the segmented regression analyses for antenatal care coverage – at least four visits (%) as a proxy for access to antenatal care.

All analyses were performed using STATA 16.0 software (StataCorp., College Station, TX, USA). Statistical significance was determined at the *P* < 0.05 level for two-sided comparisons.

### Ethics

This study used anonymised data that was retrieved from open access websites and the study was conducted according to the guidelines laid down in the Declaration of Helsinki. Approval from the Research Ethics Committee was not required for this study.

## RESULTS

### Descriptive data and time trends

**Figure 2:**
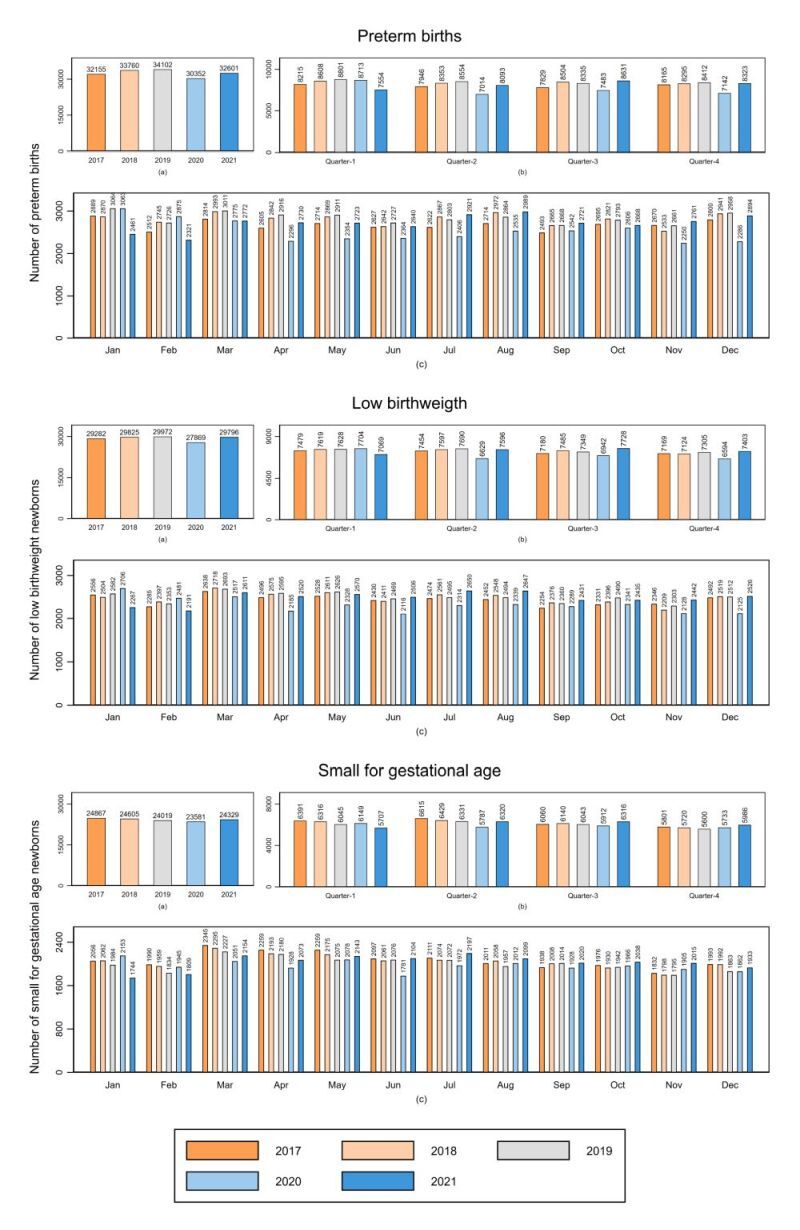
Trends annual, quarterly, and monthly of number of small vulnerable newborns in Peru, 2017–2021. Values are presented in number of absolutes. Small Vulnerable Newborn was defined as preterm birth, low birthweight and small for gestational age babies.

In Peru, there was an annual drop in the trend of absolute number of PT, LBW and SGA newborns in 2020 (30 352, 27 869, 23 581, respectively), followed by an annual increase in all of them in 2021 (32 601, 29 796, 24 329, respectively) (**Figure 2**). Additionally, there was a decline in the second, third, and fourth quarters of 2020 that continued until the first quarter of 2021. The greatest drop in PT, LBW and SGA newborns was observed in April 2020, continuing until February 2021. The Coast, the Highlands and the Amazon showed a similar trend at the national level, with larger changes in the Coast – a region with larger population – compared to other regions. Meanwhile, the Highlands and the Amazon showed an annual rise in SGA newborns from the second quarter of 2021 onwards (from 2069 to 2152 and from 1012 to 1147; respectively), predominantly in the Amazon (Figure S1 in the **Online Supplementary Document**).

**Figure 3:**
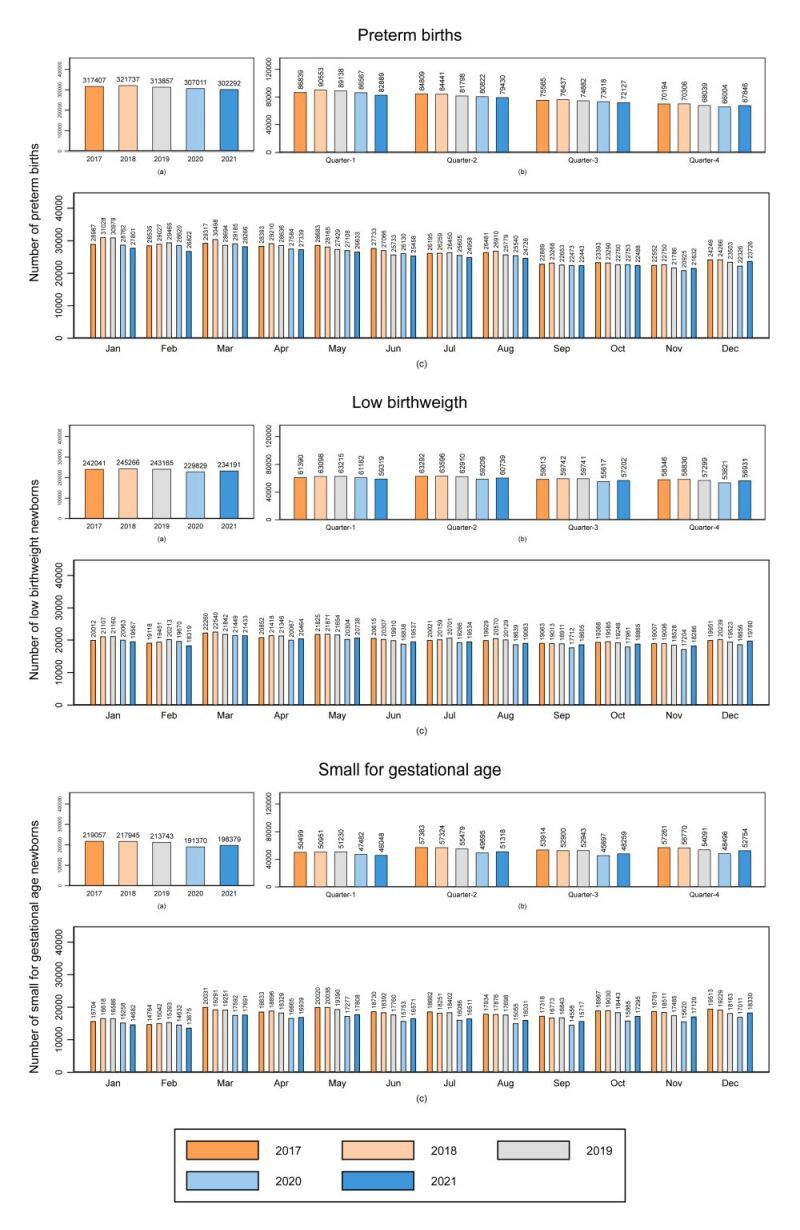
Trends annual, quarterly, and monthly of number of small vulnerable newborns in Brazil, 2017–2021. Values are presented in number of absolutes. Small Vulnerable Newborn was defined as preterm birth, low birthweight and small for gestational age babies.

In Brazil, the number of annual PT births presented a slight decrease between 2018 and 2021 (from 321 737 to 302 292) (**Figure 3**). In all years, there was an apparent decline in PT births in the third and fourth quarters of the year with the greatest drop being observed between September and November. Regarding the annual numbers of LBW and SGA, there was a slight increase in 2021. There was not much quarterly variability, but during the months of January and February the numbers of SGA were lower in all years (**Figure 3**). The same pattern was noticed in all regions of Brazil. Of note, the highest numbers of PT births, LBW and SGA were observed in the regions with larger populations (Southeast and Northeast) (Figure S2 in the **Online Supplementary Document**).

### Percent change

**Figure 4:**
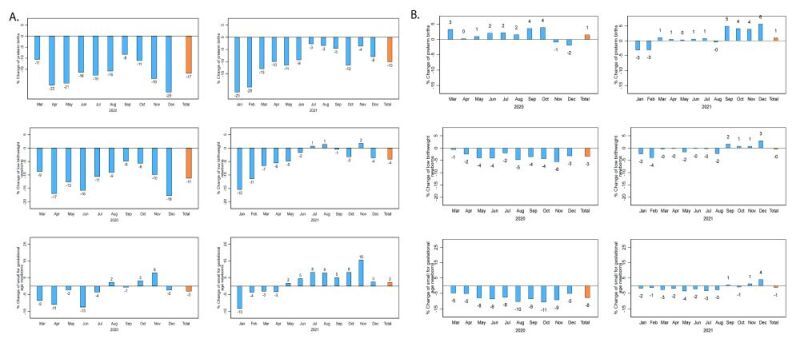
Percent change in the number of small vulnerable newborns in 2020–2021. **Panel A.** Peru. **Panel B.** Brazil. The average of January 2017–February 2020 is considered as baseline. Values are represented in percentage (%).

**Figure 5:**
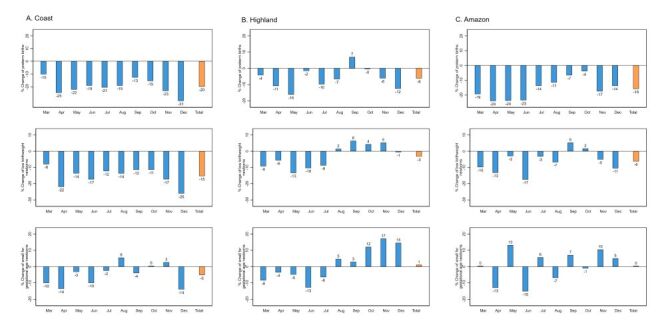
Percent change in the number of small vulnerable newborns by natural region in Peru, 2020. **Panel A.** Coast. **Panel B.** Highland. **Panel C.** Amazon. The average for January 2017–February 2020 is considered as the baseline. Values are represented in percentage (%).

The monthly percent changes in the number of PT, LBW and SGA newborns in Peru in 2020 were −17, −11, and −3% respectively. In 2021, they were −10, −4, and 2%, respectively (**Figure 4**, Panel A). At the regional level, in 2020, the Coast had the highest decline, with a percent change of −20, −15, and −5% in PT, LBW and SGA newborns, respectively. The percent change in PT, LBW and SGA newborns on the Highlands (−6, −3, and 1%, respectively) and the Amazon (−16, −6, and 0%, respectively) was relatively minor (**Figure 5**, Panels A–C). In 2021, the Coast showed the highest decrease in PT, LBW and SGA (−13, −11, and −6%, respectively), while the Highlands (−3, 6, and 14%) and the Amazon (3, 14, and 22%) showed mostly increases (Figure S3 in the **Online Supplementary Document**).

**Figure 6:**
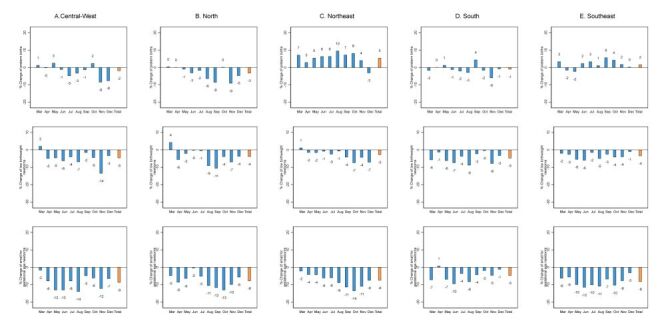
Percent change of in the number small vulnerable newborns by macroregions in Brazil, 2020. **Panel A.** Central-West. **Panel B.** North. **Panel C.** Northeast. **Panel D.** South. **Panel E.** Southeast. The average for January 2017–February 2019 is considered as the baseline. Values are represented in percentage (%).

In Brazil, the monthly percent change in the number of PT newborns was 1% in both 2020 and 2021. Newborns with LBW showed a percent change of −3 and -0%, in 2020 and 2021, respectively, while the number of SGA newborns decreased by −8% in 2020 and by −1% in 2021 (**Figure 4**, Panel B). At regional level, in 2020, PT births in the Northeast and Southeast, increased by 5 and 2%, respectively, while the Central-West, North and South regions decreased by −2, −3, and −1%, respectively (**Figure 6**, Panels A–E). Regarding LBW and SGA, all regions showed a decline in 2020, with the highest drops in LBW observed in the Central-West and South regions (−5% in both regions) and, for SGA, in the Central-West region (−8%). In 2021, the same pattern was observed, but with percent changes minor for LBW and SGA newborns (Figure S4 in the **Online Supplementary Document**).

### Segmented regression analysis

**Figure 7:**
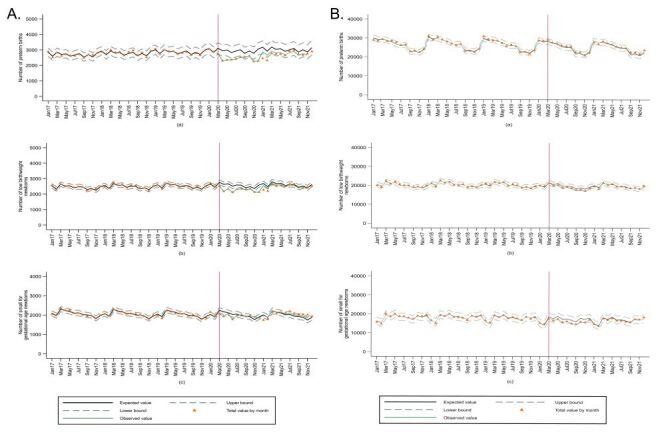
Interrupted time series analysis (monthly) for small vulnerable newborns 2017–2021. **Panel A.** Peru. **Panel B.** Brazil. The model was adjusted for each calendar month (seasonality), number of livebirths, and administrative level. Small vulnerable newborn defined as preterm births (a), low birthweight (b), and small for gestational age (c). CI – confidence interval, IRR – incidence rate ratio.

**Figure 7**, Panel A and **Table 1** show the results of the interrupted time series analysis in Peru. During the COVID-19 pandemic, the trend of PT and LBW newborns decreased, while SGA newborns increased in Peru (Table S1 in the **Online Supplementary Document**). The monthly IRR of PT births was significantly lower in March 2020–December 2020 and January 2021–December 2021 than the incidence based on the pre-pandemic period, except for July and August 2021 that were similar in both pre-pandemic and pandemic periods. Likewise, the monthly IRR of LBW was significantly lower during the whole pandemic period than the pre-pandemic period, except for December 2020, when it was lower but not significantly, while it showed a rebound, which is it was higher than the from May to December 2021. The IRR of SGA newborns has statistically increased in the period August 2020–November 2020, March 2021, and May 2021–December 2021 (**Table 1**).

**Table 1:** Incidence Rate Ratio of preterm births, low birthweight and small for gestational age newborns during COVID-19 pandemic in Peru, 2017–2021*

		Preterm birth				Low birthweight				Small for gestational age			
**Year**	**Month**	**IRR**	**95% CI**		***P*-value**	**IRR**	**95% CI**		***P*-value**	**IRR**	**95% CI**		***P*-value**
2020	March	0.93	0.92	0.93	0.000	0.96	0.95	0.96	0.000	0.98	0.97	0.99	0.000
	April	0.77	0.76	0.77	0.000	0.83	0.82	0.83	0.000	0.91	0.90	0.92	0.000
	May	0.80	0.79	0.80	0.000	0.89	0.88	0.89	0.000	0.98	0.97	0.99	0.000
	June	0.81	0.80	0.82	0.000	0.82	0.81	0.82	0.000	0.85	0.85	0.86	0.000
	July	0.84	0.82	0.85	0.000	0.91	0.91	0.92	0.000	0.97	0.97	0.98	0.000
	August	0.89	0.87	0.90	0.000	0.94	0.94	0.95	0.000	1.03	1.02	1.04	0.000
	September	0.89	0.87	0.90	0.000	0.93	0.93	0.94	0.000	1.02	1.01	1.03	0.001
	October	0.90	0.89	0.92	0.000	0.96	0.95	0.96	0.000	1.06	1.05	1.07	0.000
	November	0.77	0.75	0.78	0.000	0.86	0.86	0.87	0.000	1.03	1.02	1.04	0.000
	December	0.77	0.75	0.78	0.000	0.85	0.84	0.85	0.000	0.99	0.98	1.00	0.064
2021	January	0.81	0.80	0.82	0.000	0.88	0.88	0.89	0.000	0.90	0.89	0.91	0.000
	February	0.76	0.75	0.77	0.000	0.84	0.83	0.84	0.000	0.90	0.90	0.91	0.000
	March	0.90	0.89	0.91	0.000	0.98	0.97	0.99	0.000	1.05	1.03	1.06	0.000
	April	0.89	0.88	0.90	0.000	0.94	0.93	0.95	0.000	0.99	0.98	1.00	0.039
	May	0.90	0.88	0.91	0.000	0.96	0.96	0.97	0.000	1.02	1.01	1.03	0.000
	June	0.88	0.87	0.89	0.000	0.96	0.95	0.96	0.000	1.02	1.01	1.03	0.000
	July	0.99	0.97	1.00	0.148	1.03	1.02	1.04	0.000	1.10	1.09	1.11	0.000
	August	1.02	1.00	1.04	0.110	1.05	1.04	1.06	0.000	1.09	1.08	1.10	0.000
	September	0.93	0.90	0.95	0.000	0.98	0.97	0.99	0.000	1.08	1.07	1.10	0.000
	October	0.90	0.88	0.92	0.000	0.98	0.98	0.99	0.000	1.11	1.10	1.13	0.000
	November	0.92	0.90	0.94	0.000	0.98	0.97	0.99	0.000	1.10	1.09	1.12	0.000
	December	0.94	0.92	0.97	0.000	1.00	0.99	1.00	0.243	1.04	1.03	1.06	0.000

CI – confidence interval, IRR – incidence rate ratio

*Values represent the change in the IRR for each month during pandemic period compared to pre-pandemic period.

In the Coast, the IRR of PT births, LBW, and SGA newborns has overall decreased during COVID-19 pandemic in comparison with the expected incidences. In the Highlands, the IRR of LBW and SGA newborns were higher during COVID-19 pandemic period (August 2020–December 2021) than the pre-pandemic period. In the Amazon, the IRR of PT births (April 2021–December 2021), LBW (October 2020, March 2021-December 2021), and SGA (September 2020-December 2021) newborns has in overall increased during COVID-19 pandemic period than pre-pandemic period (Table S2 in the **Online Supplementary Document**).

**Figure 7**, Panel B and **Table 2** present the interrupted time series analysis for Brazil. During the COVID-19 pandemic, the trend of PT increased while LBW and SGA newborns decreased in Brazil (Table S3 in the **Online Supplementary Document**). The IRR of PT births was lower than the incidence based on the pre-pandemic period in most months, with an exception for March, July, August and October–2020 and January, July, August and October–2021 when there were no significant differences, or the incidences were higher than the pre-pandemic period (**Table 2**). Considering LBW, the IRR for the months during the pandemic were lower than the pre-pandemic period, except for March–2020 and March, October, and December 2021 in which there were no significant differences. Newborns with SGA exhibit a similar pattern to LBW, showing a lower IRR during the pandemic months than the pandemic period for most months, especially between June and November 2020 (**Table 2**). The IRR of PT, LBW, and SGA newborns presented overall similar patterns in most Brazilian regions in relation to the national pattern. However, regarding PT newborns, in the Northeast region the IRR was higher during the pandemic the pre-pandemic period in most months, except for April, November, and December 2020 and January, February, November, and December 2021. For May, June, September, and October 2020 and from March to June 2021, these increases in the IRR were not statistically significant (Table S4 in the **Online Supplementary Document**).

**Table 2:** Incidence Rate Ratio of preterm births, low birthweight and small for gestational age newborns during COVID-19 pandemic in Brazil, 2017–2021*

		Preterm birth				Low birthweight				Small for gestational age			
**Year**	**Month**	**IRR**	**95% CI**		***P*-value**	**IRR**	**95% CI**		***P*-value**	**IRR**	**95% CI**		***P*-value**
2020	March	1.00	0.99	1.01	0.845	1.01	1.00	1.03	0.055	1.02	0.98	1.06	0.300
	April	0.95	0.94	0.96	0.000	0.94	0.93	0.95	0.000	0.95	0.91	0.99	0.009
	May	0.96	0.95	0.97	0.000	0.96	0.94	0.97	0.000	0.96	0.92	1.00	0.072
	June	0.98	0.97	0.99	0.000	0.90	0.89	0.92	0.000	0.87	0.83	0.90	0.000
	July	1.02	1.01	1.03	0.001	0.95	0.94	0.96	0.000	0.88	0.85	0.92	0.000
	August	1.08	1.06	1.09	0.000	0.95	0.93	0.96	0.000	0.84	0.81	0.86	0.000
	September	0.98	0.96	0.99	0.005	0.92	0.91	0.93	0.000	0.82	0.80	0.85	0.000
	October	0.99	0.97	1.01	0.283	0.94	0.93	0.96	0.000	0.92	0.90	0.94	0.000
	November	0.88	0.87	0.90	0.000	0.90	0.88	0.91	0.000	0.93	0.90	0.95	0.000
	December	0.89	0.88	0.91	0.000	0.96	0.94	0.97	0.000	1.02	0.99	1.06	0.168
2021	January	1.05	1.03	1.06	0.000	0.97	0.96	0.99	0.012	0.89	0.85	0.92	0.000
	February	0.96	0.95	0.97	0.000	0.89	0.87	0.91	0.000	0.82	0.78	0.86	0.000
	March	0.98	0.97	0.99	0.001	1.02	0.99	1.04	0.164	1.04	0.99	1.10	0.140
	April	0.95	0.94	0.96	0.000	0.96	0.94	0.98	0.000	0.98	0.92	1.03	0.396
	May	0.95	0.94	0.97	0.000	0.98	0.96	1.00	0.047	1.01	0.95	1.07	0.867
	June	0.96	0.95	0.98	0.000	0.94	0.92	0.96	0.000	0.92	0.87	0.98	0.007
	July	1.01	0.99	1.02	0.507	0.96	0.94	0.99	0.001	0.92	0.87	0.97	0.002
	August	1.05	1.03	1.07	0.000	0.97	0.95	0.99	0.004	0.90	0.86	0.95	0.000
	September	0.99	0.97	1.01	0.177	0.97	0.95	0.99	0.002	0.90	0.86	0.94	0.000
	October	0.99	0.97	1.01	0.325	0.99	0.97	1.01	0.394	1.02	0.98	1.06	0.411
	November	0.92	0.91	0.94	0.000	0.96	0.93	0.98	0.000	1.03	0.99	1.08	0.170
	December	0.96	0.94	0.98	0.000	1.01	0.99	1.04	0.267	1.12	1.07	1.17	0.000

IRR – incidence rate ratio, CI – confidence interval

*Values represent the change in the IRR for each month during pandemic period compared to pre-pandemic period.

**Figure 8:**
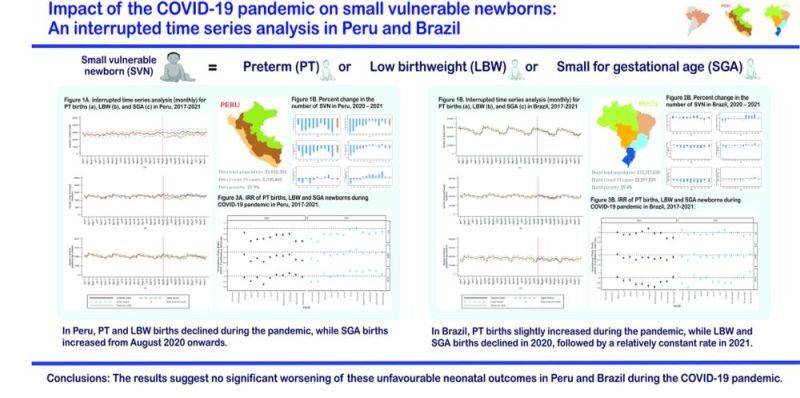
Key findings at a glance.

In the supplementary analysis, after adjustment for antenatal care coverage specifically, at least four visits (%), the findings remain similar to the main analyses (Tables S5–8 in the **Online Supplementary Document**). Finally, we summarised all our findings in **Figure 8**.

## DISCUSSSION

This study, using data from two South American countries, Peru, and Brazil, showed changes in the number of all the three phenotypes of SVN (PT, LBW or SGA) during the COVID-19 pandemic. In Peru, the PT and LBW newborns declined during the COVID-19 pandemic. However, beginning in August 2020 and extending through December 2021, we observed a rise in the number of SGA newborns at national level. Regarding Peru’s natural regions, we observed an increase in SGA babies mostly in the Amazon and the Highlands, but not in the Coast. In Brazil, PT birth exhibited a relatively steady trend during the period, with a slight increase during the pandemic years. This marginal increase is attributed to the increases observed in the Northeast and Southeast regions of the country, as the remaining regions exhibited a discreet percent reduction in both 2020 and 2021, compared to the expected values based on the preceding three years. LBW and, to a greater extent, SGA presented decreases during the months of the pandemic across all regions of Brazil in 2020 and exhibited only marginal reductions in 2021.

### Potential explanations

A plausible explanation for the declines in SVN phenotypes observed in Peru and Brazil may be associated to the lockdown and the consequent reduced risk exposures and stressors, which are often associated with SVN. It is reasonable to consider that an abrupt and significant decrease in circulating of common viral infections during lockdown, attributed to reduced social interaction, heightened hygiene measures, use of facemasks, combined with reduction in air pollution and traffic-related stresses could have resulted in the reduction of SVN newborns [2,4,47–49]. On the other hand, the primary and widely recognised cause of spontaneous PT birth is the existence of an inflammatory condition and there is supporting evidence indicating that SARS-CoV-2 infection, by inducing systemic inflammation, may initiate cervical effacement and uterine contractions through an elevation in prostaglandins, potentially contributing to preterm birth [50]. Another well-documented factor adversely affecting the incidence of SVN phenotypes is the elevated rate of caesarean sections observed in Peru [51] and Brazil [52]. Prior evidence has showed that the COVID-19 infection during pregnancy was associated to a higher risk of caesarean delivery in Brazil [53]. In parallel with this, socioeconomic disparities, unemployment and constrained access to health care may have increased during the COVID-19 pandemic in Peru [16,22,27] and in Brazil [54] and could have also indirectly impacted the occurrence of SVN phenotypes, mainly SGA.

In Peru, low health system capacities, inadequate antenatal care, poor nutritional habits and domestic violence during pregnancy could have contributed to increase adverse birth outcomes [22,30,48,55]. This situation may explain the increase of SGA newborns in Peru in 2021, mainly in both the Highlands and the Amazon. Even though Peru witnessed substantial economic growth in South America, inequalities did not substantially reduce [27]. In this study, changes observed in the newborn phenotypes might reflect the direct and indirect effect of COVID-19 pandemic, particularly in the Amazon (natural region), where the incidence of LBW and SGA has been gradually rising since 2018 and has accelerated in 2021. As our findings represent the average impact of lockdown across populations, we cannot differentiate the relative contribution of specific factors, nor whether the impact of lockdown differed between specific population subgroups. However, previous reports have identified enormous socioeconomic differences and low health system capacities per natural regions between people living in the Highlands and the Amazon compared to people living in the Coast [16,27]. Although Peru aimed at containing the COVID-19 spread with mitigation and containment measures, including prohibition of massive gatherings, implementation of curfews, and mandatory nation-wide lockdowns [56].

In Brazil, the minor changes observed in PT, LBW, and SGA newborns could be attributed to a balance between the both abovementioned factors influenced by the COVID-19 pandemic. This includes the direct impact of contract COVID-19 during pregnancy that might increase the risk of PT, LBW, and SGA newborns, and the indirect impact, related to a reduced risk of other infections and lower stress, ultimately reducing rates of SVN newborns [57]. There is also evidence of an increase in stillbirths and maternal deaths in Brazil during the pandemic [18]. Thus, the relative stability and occasional reduction in PT births observed in certain regions of Brazil (Central-West, North and South), as well as in LBW and SGA newborns may be linked to this increase in stillbirths and maternal deaths. Meanwhile, the strategy of Brazil to face the pandemic seemed unclear, with dismissing of COVID-19 as a measly cold in March 2020, and then the recommendation of just social distancing without a lockdown at national level, in contrast with the mandate of the federal government [56].

In other words, most severe circumstances of the pandemic may have resulted in tragic outcomes occurring even before birth to mothers that not received timely and quality maternity care, a hypothesis that has also been previously suggested in another study [4]. Our study calls for more research of those factors driving the exceptionally decreased of SVN in Peru and Brazil, particularly in specific regions. Finally, more studies are needed to identify those potential factors that could have contributed to increased or decreased number of SVN babies during the COVID-19 pandemic using longitudinal studies.

Furthermore, our study calls for more research of factors driving the exceptionally decreased of SVN in Peru, particularly in the Coast, and explore in more detail the factors contributing to the slight changes in SVN phenotypes in Brazil. Another well-documented factor adversely affecting the incidence of SVN phenotypes is the elevated rate of caesarean sections observed in Peru [51] and Brazil [52]. Moreover, previous evidence showed that the COVID-19 infection during pregnancy was associated to a higher risk of caesarean delivery in Brazil [53].

### Strengths and limitations

Strengths of our study include the use of two large national birth registries in Peru and Brazil covering the period 2017–2021. The rich databases allowed the analysis of time trend series at national and regional levels with the incorporation of a consistent baseline comparison period of three years. Moreover, we used robust statistical approaches for estimating monthly variations that considered historical trends, seasonal effects, and changes in the population (number of livebirths). Nevertheless, we acknowledge some limitations. Misclassification bias due to differences in registration procedure among regions might not be excluded, despite gestational age was estimated based on ultrasound or last menstrual period, confirmed by physical examination. These issues may either overestimate or underestimate the count of premature babies [39]. On the other hand, it is important to highlight that data entry in the national registration systems followed standard procedures and was performed by health professional using national guidelines [35,38,58]. The level of data completeness from the health services could also be a limitation [59]. However, the information coverage of the Peru and Brazilian health systems was considered high and adequate, which provides greater reliability regarding data integrity [4,39]. The reliance on national health information systems may introduce bias due to incomplete or delayed data entry during the COVID-19 pandemic period, despite the coverage information consistently remained above 81% throughout the study period in Peru and in Brazil [4,23,60]. The observed changes in the SVN trends should be interpreted with caution between the pre- and post-pandemic periods, and not be considered as direct impact of the COVID-19 pandemic, since other potential causes may influence these outcomes.

### Public health implications

The pandemic emphasised the importance of allocating resources to robust health care systems capable of effectively addressing crises while continuing to provide essential services, mainly in vulnerable populations such as pregnant women and newborn. Socioeconomic and geographic differences in health care access may still result in insufficient and inadequate maternal and neonatal health care. Moreover, socioeconomic, and geographic disparities between Peru and Brazil and their regions may differ according to the well-being indicators. Even though our study showed slightly reductions in SVN phenotypes during the COVID-19 pandemic in both countries, Peru and Brazil have reported unfavourable maternal and neonatal outcomes, such as an increase in stillbirths and maternal mortality during the pandemic [18]. Therefore, ensuring continuous access to antenatal care may prevent or identify perinatal complications and avoid stillbirths and avoidable maternal deaths. As a result, sustained efforts to protect maternal, newborn and child health (MNCH) services must remain highly monitored, particularly during crises, such as COVID-19 pandemic.

As there are clear differences across regions and countries, it would be key to identify and implement tailored strategies to better support pregnant women during and beyond the COVID-19 pandemic. This recommendation is underscored by the rise in SGA babies in Peru since August 2020, particularly in the Highlands and the Amazon – regions characterised for socioeconomic disparities and limited access to health care. Prior studies consistently shown that SGA babies are associated with adverse long-term health outcomes, highlighting the need for region-specific interventions to address these disparities.

To foster continuous improvement, several strategies can be pursued. These include investing in Primary Healthcare Infrastructure by allocating resources towards strengthening primary health care and expanding the health care workforce to improve the health care access. Furthermore, it is essential to explore new models of care, as innovative care models and technologies that can enhance the efficiency, effectiveness, and access of health care delivery and finally to improve multidisciplinary collaboration and enhance the availability of health care professionals particularly in underserved regions.

## CONCLUSIONS

Our findings suggest that the major changes in PT, LBW, and SGA newborns phenotypes occurred in 2020 in both Peru and Brazil. Specifically, both countries exhibited a decrease in LBW during the pandemic when comparing to the expected numbers, while Peru displayed a relative rise in SGA since August 2020 and Brazil showed a slight increase in PT births.

The impact of PT births, LBW and SGA on the morbidity and mortality of newborns, along with the potential long-lasting consequences and the substantial costs for the health care systems, underscores the importance of disentangle information about their drivers of this occurrence. This data is crucial for the planning and organisation of health care services, particularly for the most vulnerable groups. Additional research is warranted to monitor future trend shifts during subsequent pandemic years beyond 2021 and investigate potential underlying mechanisms.

## Additional material


Online Supplementary Document
