# Supplementary material for: Impact of the COVID-19 pandemic on small vulnerable newborns: an interrupted time series analysis in Peru and Brazil
Source: J Glob Health. 2025 Jan 31;15:04026. doi: 10.7189/jogh.15.04026 (PMC11783137; doi:10.7189/jogh.15.04026)
Supplement: Online Supplementary Document [file jogh-15-04026-s001.pdf]

Supplementary Material

Impact of the COVID-19 pandemic on small vulnerable newborns: An interrupted time series analysis in Peru and Brazil

Kim N Cajachagua-Torres*[1,2,3] (https://orcid.org/0000-0002-0213-6935), Mariana Otero Xavier*[4] (http://orcid.org/0000-0001-8791-3520), Hugo G Quezada-Pinedo[2,3,5] (https://orcid.org/0000-0002-0641-7718), Carlos A Huayanay-Espinoza[3] (https://orcid.org/0000-0002-8462-3218), Alvaro Gonzalo Oviedo Rios[3] (https://orcid.org/0009-0005-0413-7252), Agbessi Amouzou[6] (http://orcid.org/0000-0002-6262-3866), Abdoulaye Maïga[6] (http://orcid.org/0000-0002-0475-1106), Nadia Akseer[6] (http://orcid.org/0000-0002-3802-7298), Alicia Matijasevich[4†] (http://orcid.org/0000-0003-0060-1589), Luis Huicho[3,7†] (http://orcid.org/0000-0002-5272-5885)

* First authors contributed equally

† Joint senior authorship

[1]Department of Pediatrics, New York University Grossman School of Medicine, New York, NY, USA

[2]Department of Pediatrics, Erasmus MC, University Medical Centre Rotterdam, Rotterdam, The Netherlands

[3]Centro de Investigación en Salud Materna e Infantil and Centro de Investigación para el Desarrollo Integral y Sostenible, Universidad Peruana Cayetano Heredia, Lima, Peru.

[4]Departamento de Medicina Preventiva, Faculdade de Medicina FMUSP, Universidade de São Paulo, São Paulo, Brasil

[5]Department of Population Health Sciences, Duke University School of Medicine, Durham, NC, USA

[6]Department of International Health, Johns Hopkins University Bloomberg School of Public Health, Baltimore, Maryland, USA

[7]Facultad de Medicina, Universidad Peruana Cayetano Heredia, Lima, Peru

Content

- **Figure S1.** Annual, quarterly, and monthly number of small vulnerable newborns by natural regions (Coast (A), Highlands (B), and Amazon (C)) in Peru, 2017-2021.
- **Figure S2.** Annual, quarterly, and monthly number of small vulnerable newborns by region (Central West (A), North (B), Northeast (C), South (D), and South East (E)) in Brazil, 2017-2021.
- **Figure S3.** Percent change of small vulnerable newborns by natural regions in Peru, 2021.
- **Figure S4.** Percent change of small vulnerable newborns by region in Brazil, 2021.
- **Table S1.** Percent change in small vulnerable newborn by month from March 2020 to December 2021 in Peru, compared to the expected numbers based on the preceding three years.
- **Table S2.** Incidence Rate Ratio of preterm births, low birthweight and small for gestational age newborns during COVID-19 by natural regions in Peru, 2017-2021.
- **Table S3.** Percent change in small vulnerable newborn by month from March 2020 to December 2021 in Brazil, compared to the expected numbers based on the preceding three years.
- **Table S4.** Incidence Rate Ratio of preterm births, low birthweight and small for gestational age newborns during COVID-19 by region in Brazil, 2017-2021.
- **Table S5.** Percent change in small vulnerable newborn by month from March 2020 to December 2021 after adjustment for antenatal care at least four times in Peru, compared to the expected numbers based on the preceding three years.
- **Table S6.** Percent change in small vulnerable newborn by month from March 2020 to December 2021 after adjustment for antenatal care at least four times in Brazil, compared to the expected numbers based on the preceding three years.
- **Table S7.** Incidence Rate Ratio of preterm births, low birthweight and small for gestational age newborns during COVID-19 by natural regions after adjustment for antenatal care at least four times in Peru, 2017-2021.
- **Table S8.** Incidence Rate Ratio of preterm births, low birthweight and small for gestational age newborns during COVID-19 by region after adjustment for antenatal care at least four times in Brazil, 2017-2021.
- **Appendix S1.** Abstract in Spanish and Portuguese

Figure S1. Annual, quarterly, and monthly number of small vulnerable newborns by natural regions in Peru, 2017-2021.

A. Coast

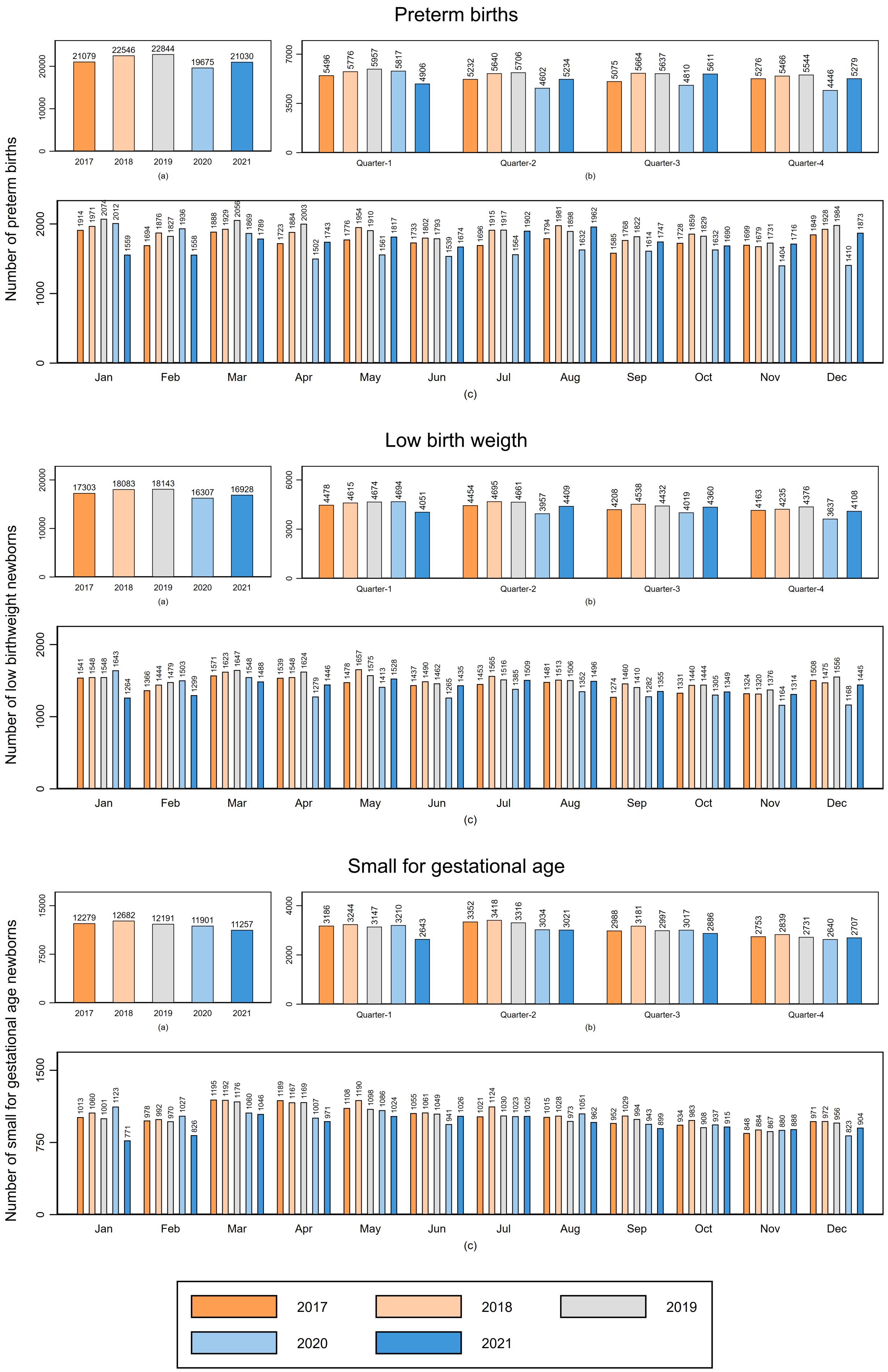

Notes: Results are presented in number of absolutes. Small Vulnerable Newborn was defined as preterm birth, low birthweight and small for gestational age babies. Peru is geographically divided in three natural regions (Coast, Highlands and Amazon). The Coast (A) is extended along the Pacific Ocean.

B. Highlands

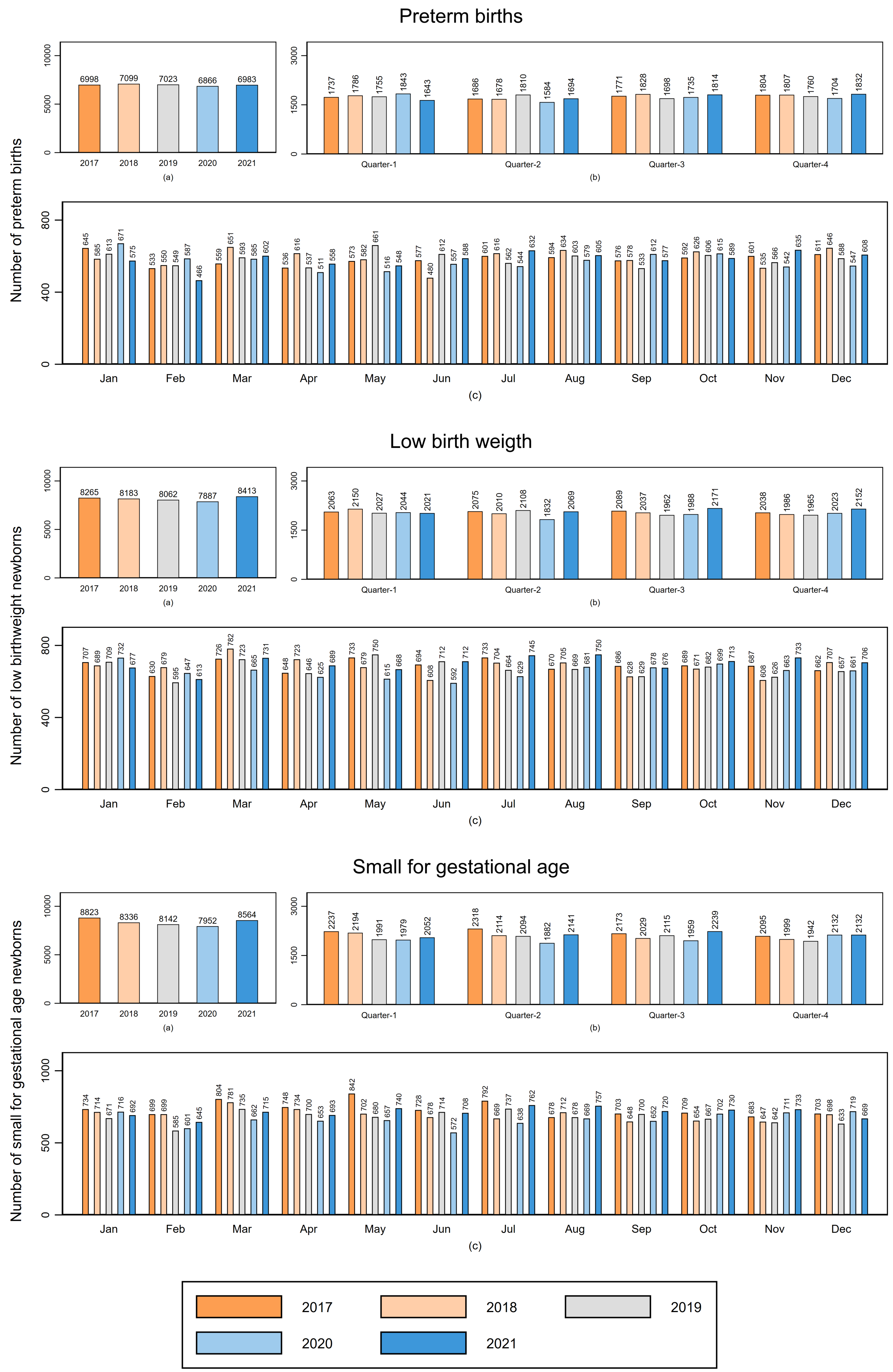

Notes: Results are presented in number of absolutes. Small Vulnerable Newborn was defined as preterm birth, low birthweight and small for gestational age babies. Peru is geographically divided in three natural regions (Coast, Highlands and Amazon). The Highlands (B) are extended surrounding the Andes.

C. Amazon

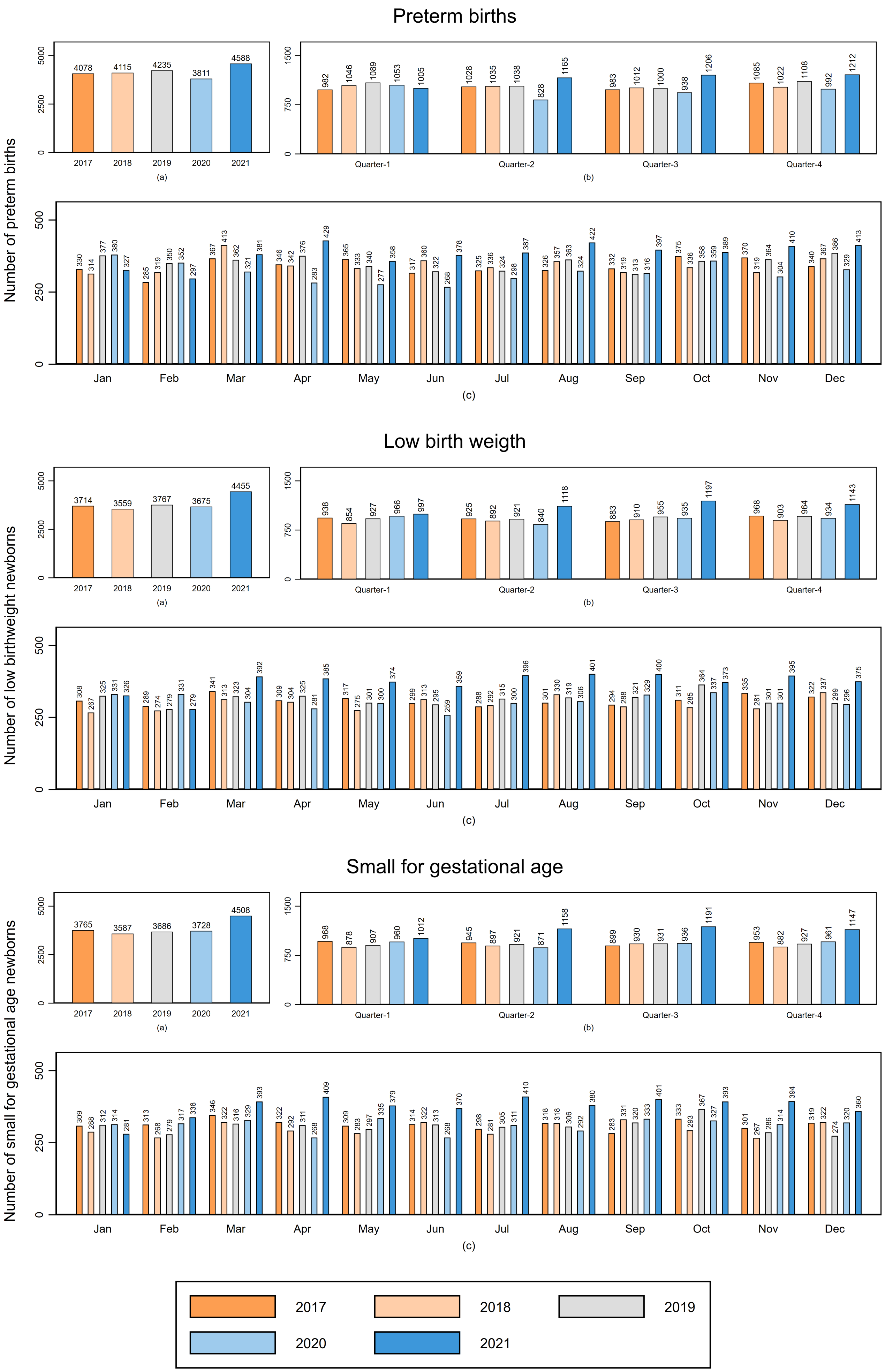

Notes: Results are presented in number of absolutes. Small Vulnerable Newborn was defined as preterm birth, low birthweight and small for gestational age babies. Peru is geographically divided in three natural regions (Coast, Highlands and Amazon). The Amazon (C) is extended around the Amazon rainforest.

Figure S2. Annual, quarterly, and monthly number of small vulnerable newborns by region in Brazil, 2017-2021

A. Central-West

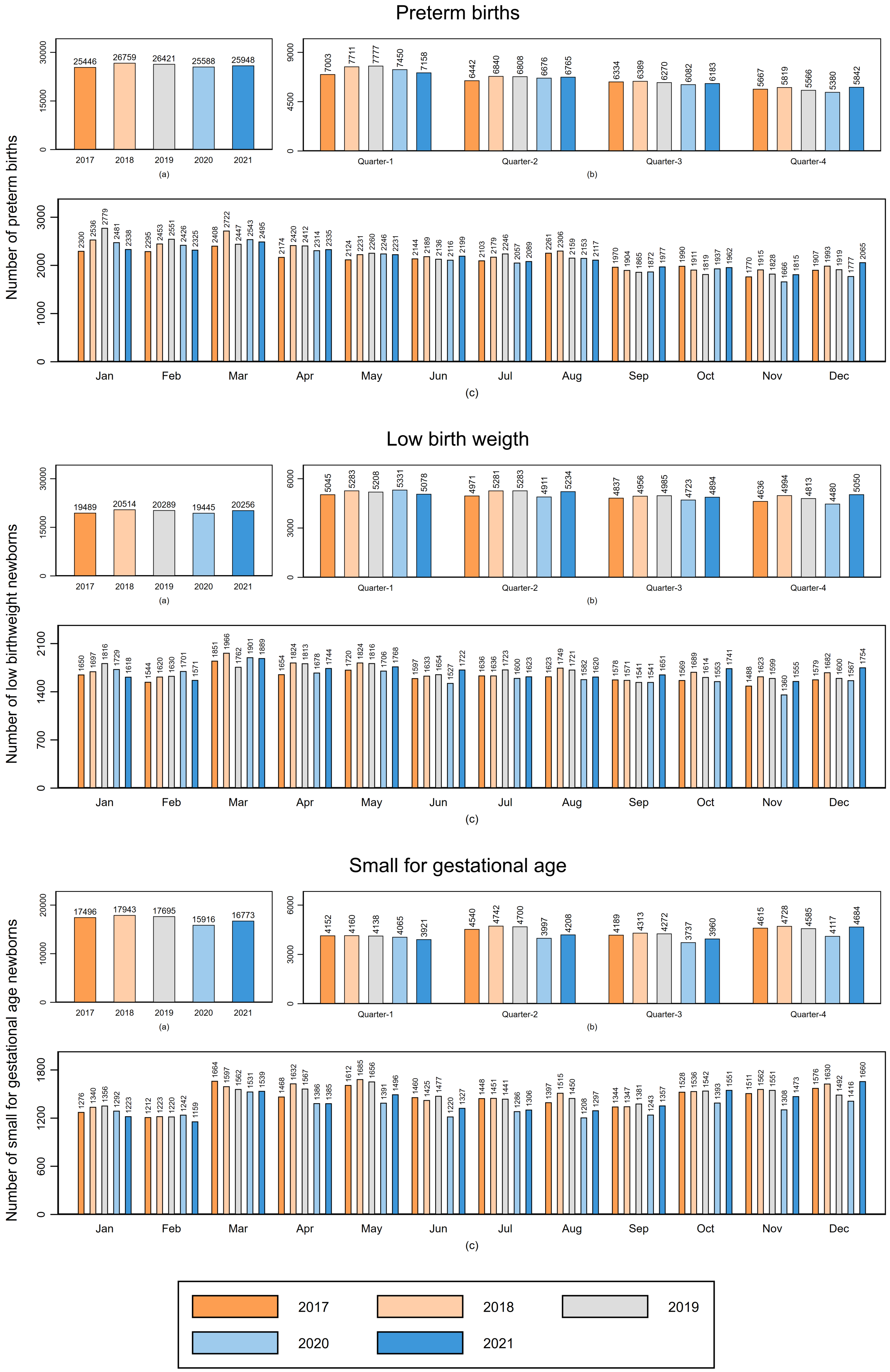

Notes: Results are presented in number of absolutes. Small Vulnerable Newborn was defined as preterm birth, low birthweight and small for gestational age babies. Brazil is geographically divided in five regions (Central-West (A), North (B), Northeast (C), South (D) and Southeast (E)).

B. North

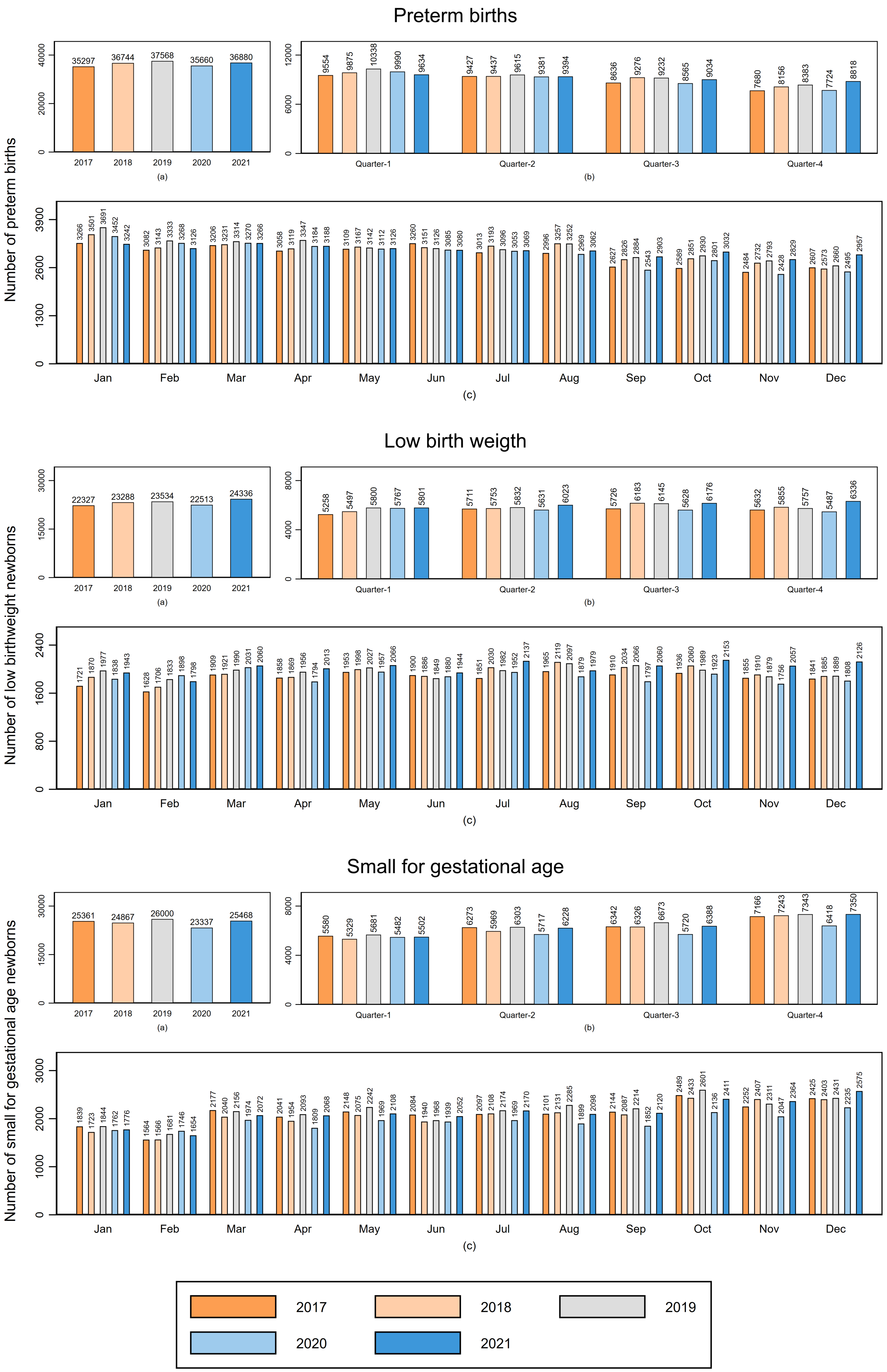

Notes: Results are presented in number of absolutes. Small Vulnerable Newborn was defined as preterm birth, low birthweight and small for gestational age babies. Brazil is geographically divided in five regions (Central-West (A), North (B), Northeast (C), South (D) and Southeast (E)).

C. Northeast

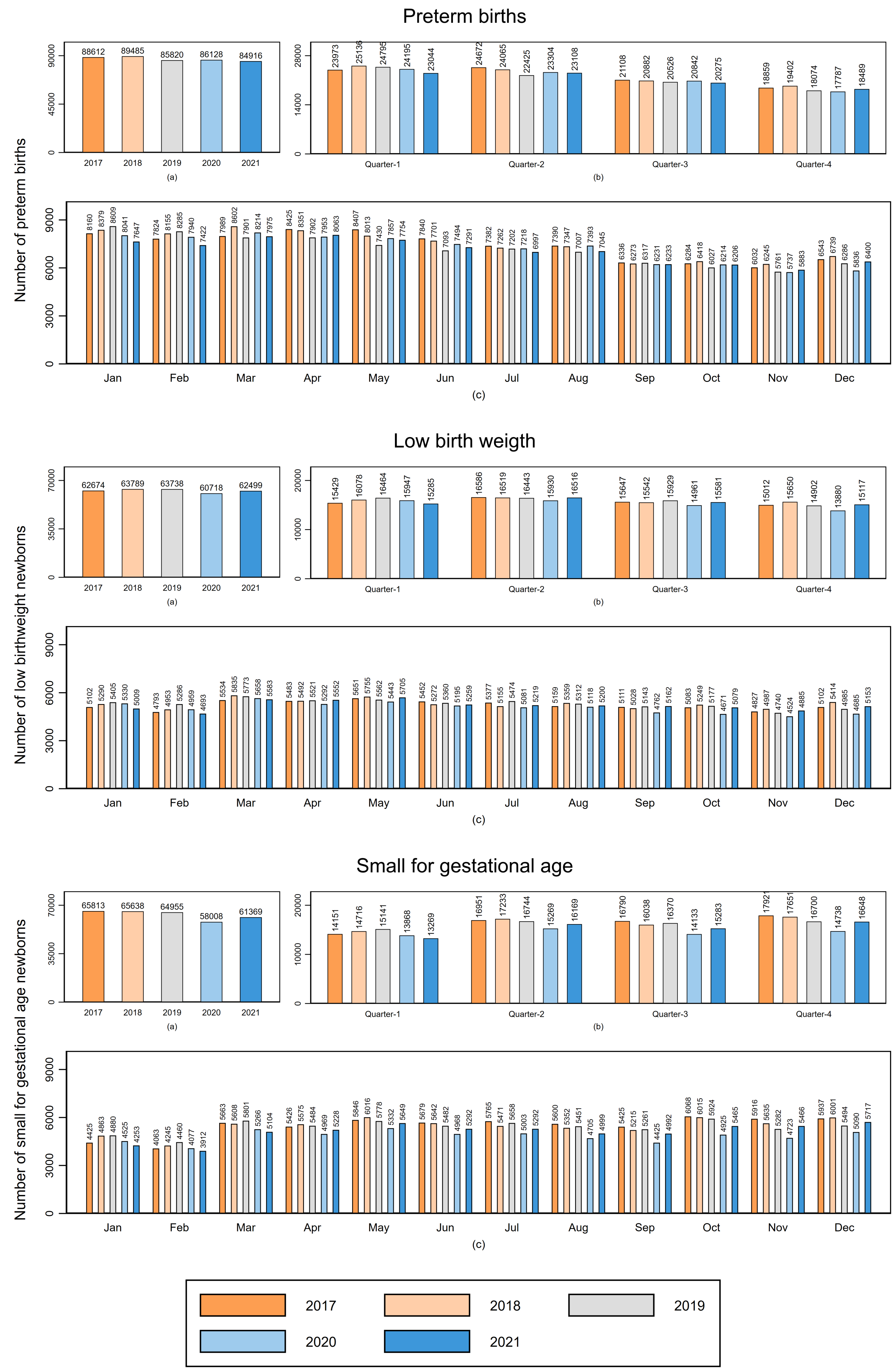

Notes: Results are presented in number of absolutes. Small Vulnerable Newborn was defined as preterm birth, low birthweight and small for gestational age babies. Brazil is geographically divided in five regions (Central-West (A), North (B), Northeast (C), South (D) and Southeast (E)).

D. South

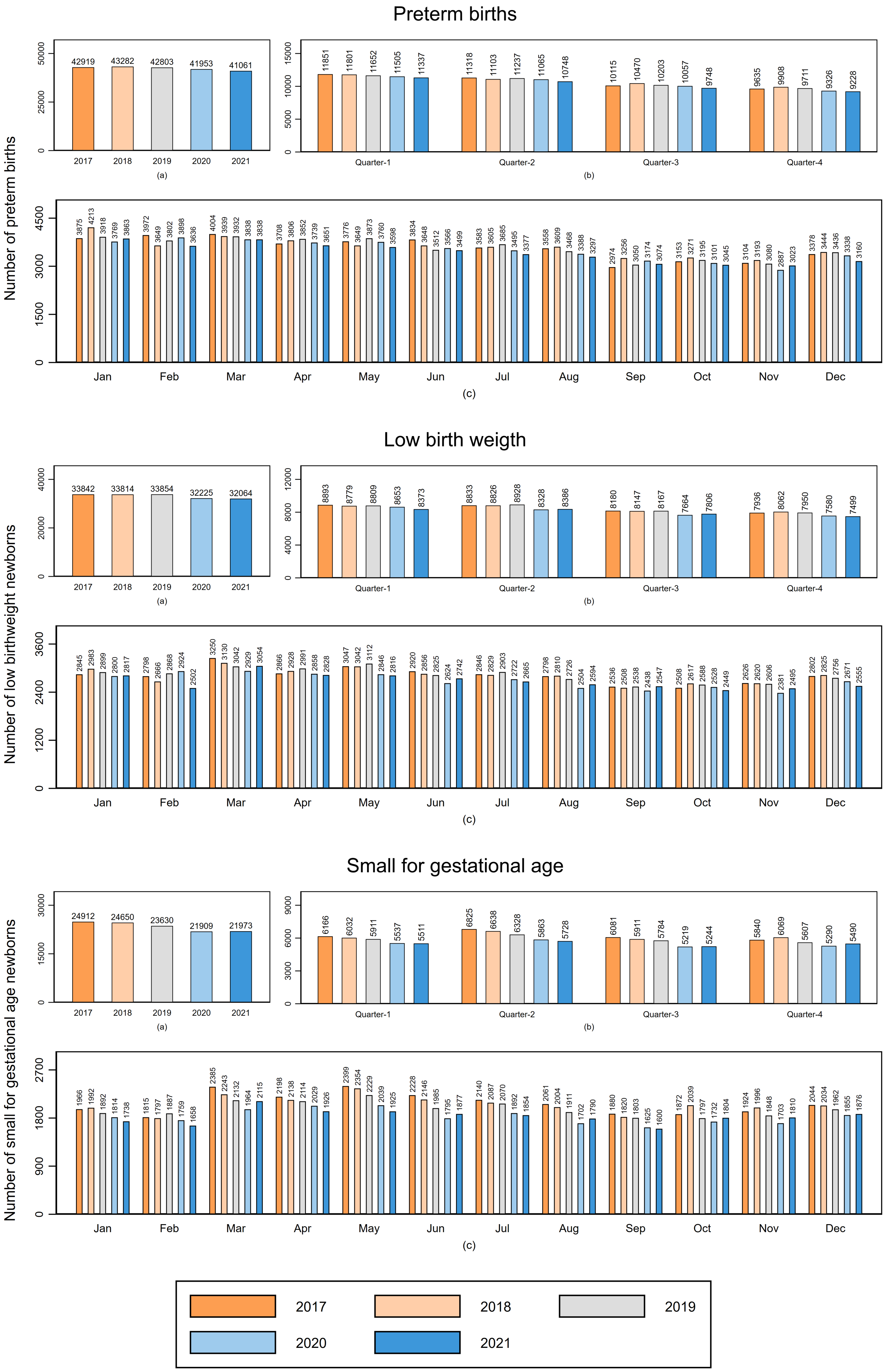

Notes: Results are presented in number of absolutes. Small Vulnerable Newborn was defined as preterm birth, low birthweight and small for gestational age babies. Brazil is geographically divided in five regions (Central-West (A), North (B), Northeast (C), South (D) and Southeast (E)).

E. Southeast

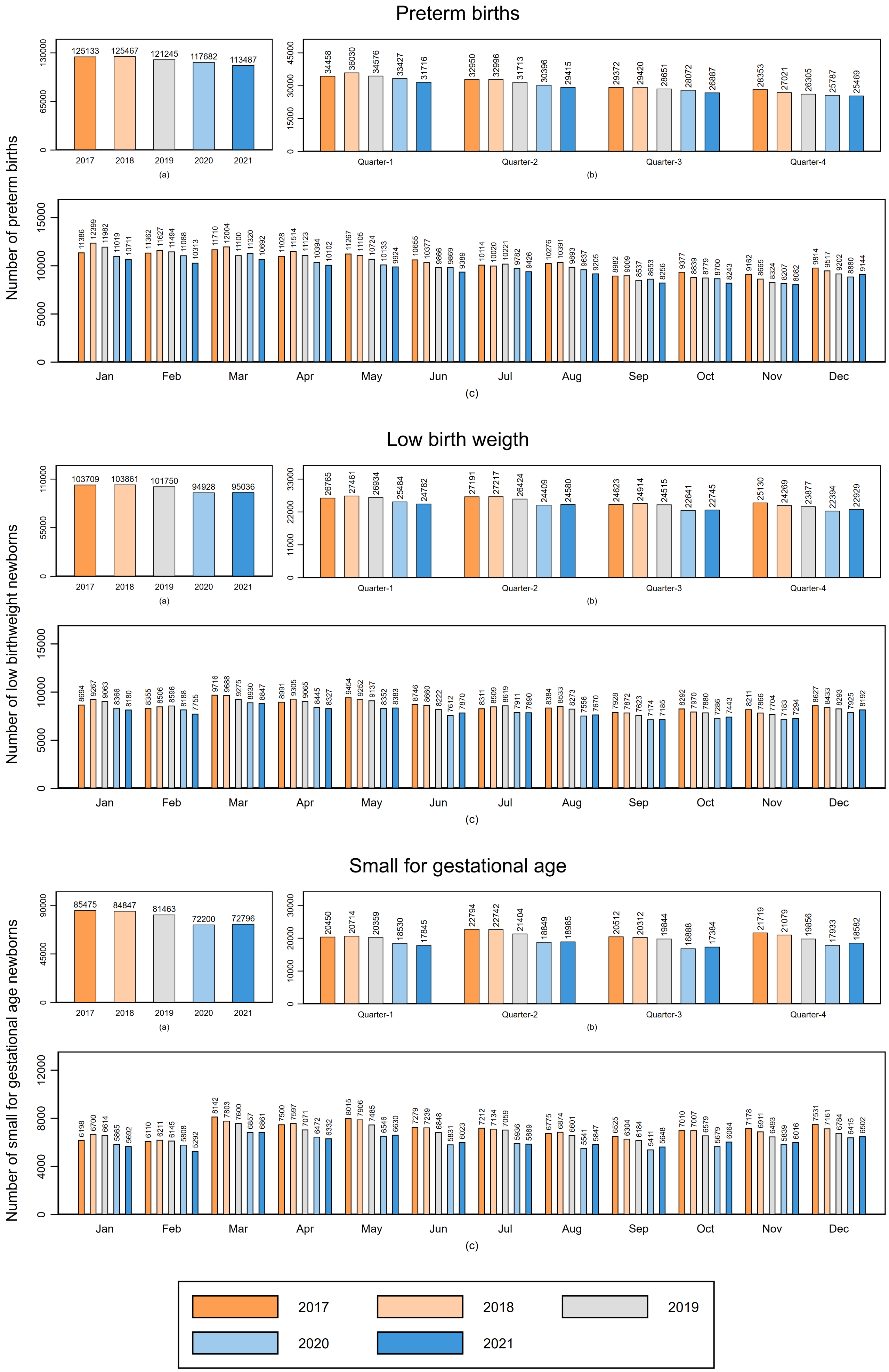

Notes: Results are presented in number of absolutes. Small Vulnerable Newborn was defined as preterm birth, low birthweight and small for gestational age babies. Brazil is geographically divided in five regions (Central-West (A), North (B), Northeast (C), South (D) and Southeast (E)).

Figure S3. Percent change of small vulnerable newborns by natural region in Peru, 2021.

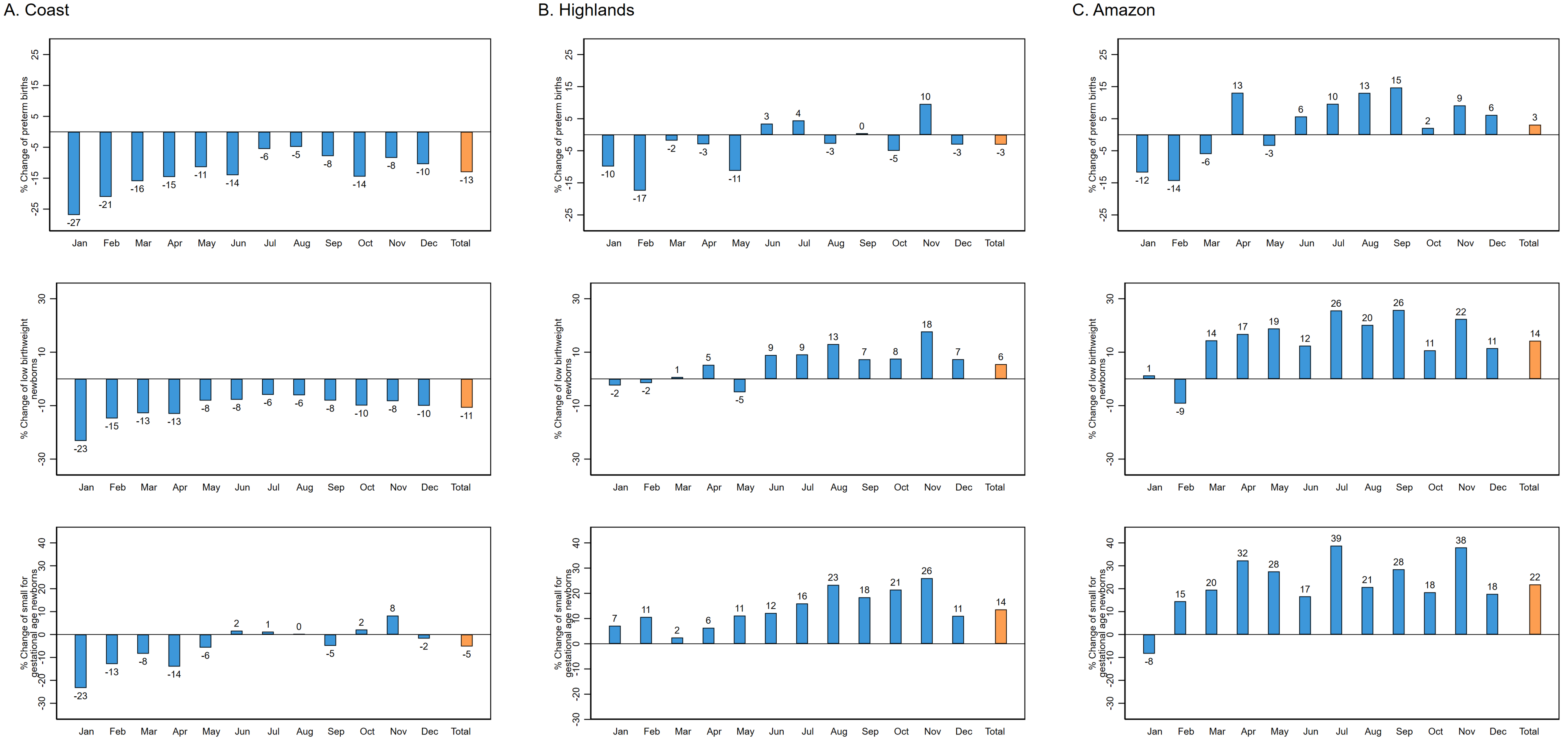

Notes: The graph considered the average Jan-2017-Feb-2020 as baseline. Values are represented in percentage (%). Peru is geographically divided in three natural regions (Coast (A), Highlands (B) and Amazon (C)).

Figure S4. Percent change of small vulnerable newborns by natural region in Brazil, 2021.

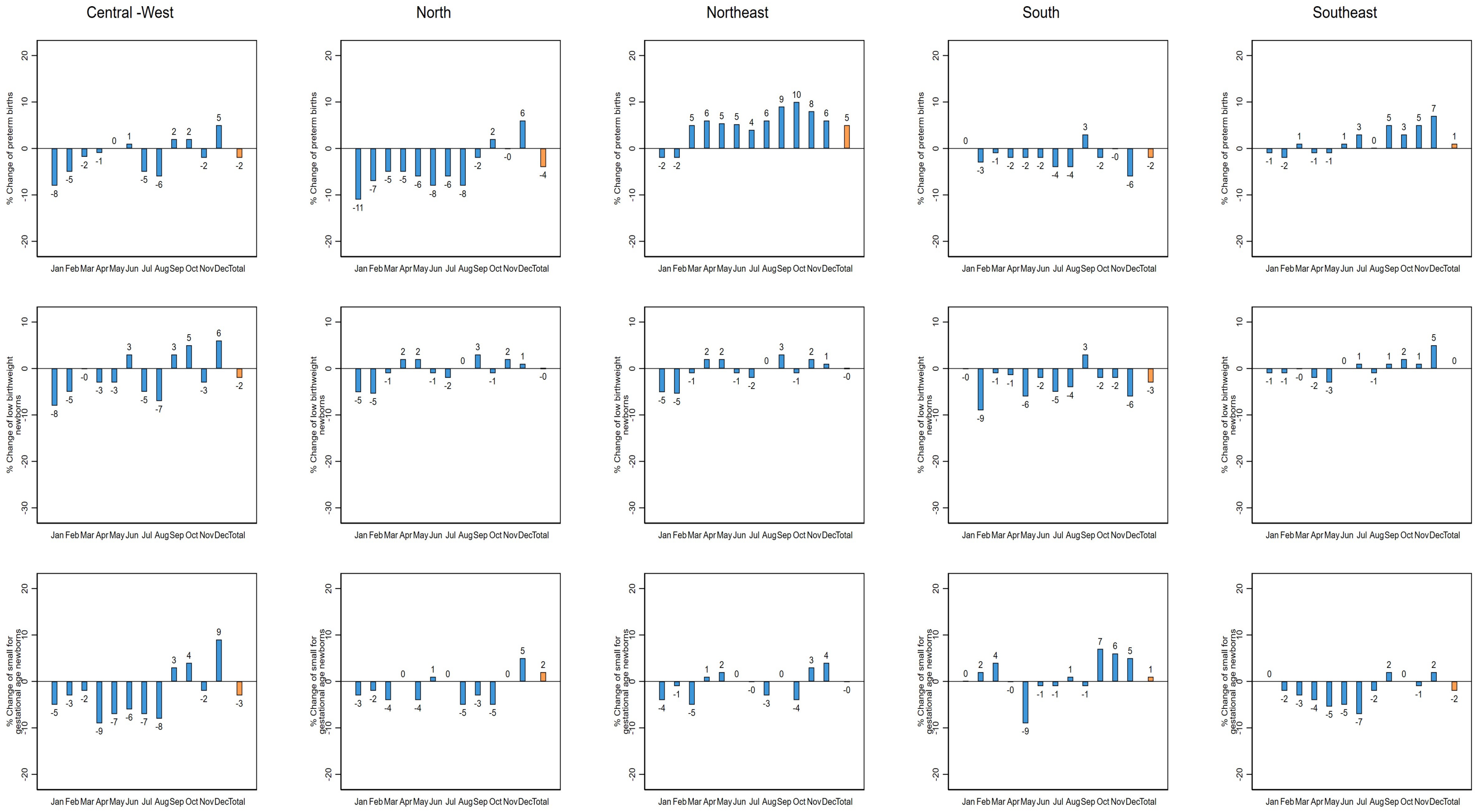

Notes: The graph considered the average Jan-2017-Feb-2020 as baseline. Values are represented in percentage (%). Brazil is geographically divided in five regions (Central-West (A), North (B), Northeast (C), South (D) and Southeast (E)).

Table S1. Percent change in small vulnerable newborn by month from March 2020 to December 2021 in Peru, compared to the expected numbers based on the preceding three years.

Timepoints (month year)	Observed	Expected	Observed 95% CI		Expected 95% CI		% change
**Preterm births**							
Mar 2020	2775	3103	2526.26	3143.63	2739.40	3465.80	-0.11
Apr 2020	2296	2951	2290.82	2912.28	2583.31	3318.83	-0.22
May 2020	2354	2995	2337.01	2960.55	2623.09	3366.34	-0.21
Jun 2020	2364	2828	2213.30	2839.91	2452.60	3204.38	-0.16
Jul 2020	2406	2925	2282.44	2936.75	2535.86	3314.56	-0.18
Aug 2020	2535	3013	2390.95	3024.88	2628.31	3398.15	-0.16
Sep 2020	2542	2772	2210.55	2846.98	2382.96	3161.09	-0.08
Oct 2020	2606	2933	2345.16	2985.46	2539.03	3326.50	-0.11
Nov 2020	2250	2784	2143.23	2786.97	2386.21	3182.75	-0.19
Dec 2020	2286	3063	2259.71	3006.24	2660.37	3465.59	-0.25
**2020 TOTAL**	24414	29368					-0.17
Jan 2021	2461	3176	2087.98	2834.02	2768.77	3582.66	-22.51
Feb 2021	2321	2918	1946.42	2695.58	2506.58	3329.81	-20.46
Mar 2021	2772	3185	2395.82	3148.18	2762.84	3606.67	-12.96
Apr 2021	2730	3033	2351.80	3108.20	2605.69	3459.52	-9.98
May 2021	2723	3076	2343.49	3102.51	2644.98	3507.74	-11.49
Jun 2021	2640	2911	2258.90	3021.11	2474.49	3346.68	-9.30
Jul 2021	2921	3005	2531.08	3310.92	2557.85	3451.48	-2.78
Aug 2021	2989	3096	2603.99	3374.01	2649.41	3541.60	-3.44
Sep 2021	2721	2854	2334.75	3107.26	2403.04	3304.25	-4.65
Oct 2021	2668	3015	2279.50	3056.50	2559.53	3471.27	-11.52
Nov 2021	2761	2866	2370.90	3151.11	2405.79	3327.11	-3.68
Dec 2021	2894	3145	2502.49	3285.51	2679.68	3610.35	-7.98
**2021 TOTAL**	32601	36279					-10.14
**Low birthweight**							
Mar 2020	2517	2759	2307.12	2873.53	2607.04	2910.31	-8.76

Apr 2020	2185	2631	2125.25	2697.75	2478.44	2783.15	-16.95
May 2020	2328	2664	2183.38	2760.65	2512.14	2815.52	-12.61
Jun 2020	2116	2513	2014.01	2596.77	2360.85	2664.16	-15.78
Jul 2020	2314	2589	2108.81	2713.23	2419.57	2758.15	-10.62
Aug 2020	2339	2574	2109.79	2704.41	2421.47	2725.98	-9.12
Sep 2020	2289	2406	1968.60	2568.45	2253.84	2557.23	-4.84
Oct 2020	2341	2482	2035.43	2641.43	2329.55	2633.66	-5.67
Nov 2020	2128	2362	1889.44	2501.36	2209.79	2513.93	-9.90
Dec 2020	2125	2583	2052.08	2669.52	2431.67	2734.87	-17.74
**2020 TOTAL**	22682	25561					-11.26
Jan 2021	2267	2681	1924.01	2609.99	2530.39	2832.37	-15.45
Feb 2021	2191	2474	1845.23	2536.77	2323.19	2625.21	-11.45
Mar 2021	2611	2796	2262.42	2959.58	2632.75	2958.66	-6.61
Apr 2021	2520	2669	2168.30	2871.70	2505.20	2832.36	-5.57
May 2021	2570	2702	2215.65	2924.35	2538.57	2864.73	-4.87
Jun 2021	2506	2550	2148.79	2863.21	2386.64	2712.62	-1.71
Jul 2021	2650	2630	2285.23	3014.77	2454.56	2805.56	0.76
Aug 2021	2647	2611	2283.55	3010.45	2446.78	2774.37	1.40
Sep 2021	2431	2443	2064.88	2797.12	2280.35	2606.43	-0.51
Oct 2021	2435	2518	2065.53	2804.47	2354.05	2681.73	-3.29
Nov 2021	2442	2399	2069.60	2814.40	2235.63	2562.73	1.78
Dec 2021	2526	2620	2150.78	2901.22	2457.54	2783.42	-3.61
**2021 TOTAL**	29796	31093					-4.17
**Small for gestational age**							
Mar 2020	2051	2246	1898.93	2448.95	2109.22	2383.54	-8.70
Apr 2020	1928	2168	1805.60	2363.19	2029.95	2305.58	-11.06
May 2020	2078	2127	1809.06	2373.24	1989.60	2264.02	-2.29
Jun 2020	1781	2036	1662.56	2233.89	1898.42	2172.77	-12.51
Jul 2020	1972	2047	1707.03	2297.69	1894.11	2200.37	-3.68
Aug 2020	2012	1966	1660.76	2247.13	1828.39	2103.83	2.33
Sep 2020	1928	1944	1619.72	2213.10	1806.64	2081.07	-0.82

Oct 2020	1966	1907	1597.43	2198.55	1769.52	2044.60	3.09
Nov 2020	1905	1766	1472.63	2081.32	1628.40	1903.50	7.87
Dec 2020	1862	1907	1563.93	2179.91	1769.48	2043.74	-2.34
**2020 TOTAL**	19483	20113					-3.13
Jan 2021	1744	2010	1408.85	2079.16	1873.62	2146.77	-13.24
Feb 2021	1809	1880	1470.24	2147.76	1742.92	2016.11	-3.75
Mar 2021	2154	2224	1811.59	2496.41	2076.60	2371.40	-3.15
Apr 2021	2073	2147	1726.70	2419.30	1998.68	2294.61	-3.43
May 2021	2143	2105	1793.16	2492.84	1957.96	2252.98	1.78
Jun 2021	2104	2013	1750.46	2457.54	1865.91	2160.77	4.50
Jul 2021	2197	2030	1836.25	2557.75	1871.63	2189.12	8.21
Aug 2021	2099	1943	1737.64	2460.36	1795.34	2091.65	8.00
Sep 2021	2020	1923	1655.05	2384.95	1775.09	2070.04	5.07
Oct 2021	2038	1884	1668.96	2407.04	1735.50	2031.90	8.19
Nov 2021	2015	1744	1642.17	2387.83	1596.02	1891.89	15.54
Dec 2021	1933	1884	1556.46	2309.54	1737.08	2031.86	2.58
**2021 TOTAL**	24329	23788					2.28

Notes: The model was adjusted for each calendar month (seasonality), number of livebirths, and administrative level. Small Vulnerable Newborn was defined as preterm birth, low birthweight and small for gestational age babies Abbreviations: 95% CI – 95% confidence interval.

Table S2. Incidence Rate Ratio (IRR) of preterm births, low birthweight and small for gestational age newborns during COVID-19 by natural regions in Peru, 2017-2021.

		Preterm births				Low birthweight				Small for gestational age			
**Year**	**Month**	**IRR**	**95% CI**		**p-value**	**IRR**	**95% CI**		**p-value**	**IRR**	**95% CI**		**p-value**
**A. Coast**													
2020	March	0.92	0.90	0.93	0.000	0.95	0.94	0.96	0.000	0.95	0.93	0.96	0.000
	April	0.74	0.73	0.75	0.000	0.78	0.77	0.79	0.000	0.88	0.86	0.89	0.000
	May	0.78	0.77	0.79	0.000	0.87	0.85	0.88	0.000	0.95	0.93	0.97	0.000
	June	0.78	0.77	0.79	0.000	0.79	0.78	0.80	0.000	0.85	0.83	0.86	0.000
	July	0.81	0.80	0.82	0.000	0.89	0.88	0.91	0.000	0.97	0.95	0.99	0.001
	August	0.85	0.84	0.87	0.000	0.90	0.89	0.91	0.000	1.05	1.04	1.07	0.000
	September	0.85	0.83	0.86	0.000	0.87	0.86	0.88	0.000	0.99	0.98	1.00	0.194
	October	0.85	0.83	0.87	0.000	0.89	0.88	0.90	0.000	1.01	1.00	1.02	0.102
	November	0.72	0.70	0.73	0.000	0.78	0.77	0.79	0.000	0.95	0.94	0.96	0.000
	December	0.70	0.69	0.72	0.000	0.76	0.76	0.77	0.000	0.86	0.85	0.87	0.000
2021	January	0.76	0.74	0.78	0.000	0.80	0.79	0.81	0.000	0.76	0.75	0.78	0.000
	February	0.75	0.73	0.76	0.000	0.80	0.78	0.81	0.000	0.77	0.76	0.79	0.000
	March	0.85	0.83	0.87	0.000	0.89	0.88	0.91	0.000	0.93	0.92	0.95	0.000
	April	0.83	0.81	0.85	0.000	0.86	0.84	0.87	0.000	0.85	0.83	0.87	0.000
	May	0.87	0.86	0.89	0.000	0.92	0.90	0.93	0.000	0.89	0.87	0.92	0.000
	June	0.82	0.81	0.84	0.000	0.88	0.86	0.90	0.000	0.92	0.90	0.95	0.000
	July	0.95	0.93	0.97	0.000	0.95	0.94	0.97	0.000	0.97	0.95	0.99	0.012
	August	0.99	0.97	1.02	0.508	0.97	0.96	0.99	0.000	0.96	0.94	0.98	0.001
	September	0.89	0.86	0.91	0.000	0.90	0.89	0.91	0.000	0.94	0.93	0.96	0.000
	October	0.85	0.83	0.87	0.000	0.90	0.89	0.91	0.000	0.99	0.97	1.00	0.095
	November	0.85	0.82	0.87	0.000	0.86	0.85	0.88	0.000	0.95	0.94	0.97	0.000
	December	0.90	0.87	0.93	0.000	0.92	0.91	0.94	0.000	0.94	0.93	0.96	0.000

**B. Highlands**													
2020	March	0.98	0.97	1.00	0.048	0.97	0.96	0.98	0.000	0.99	0.98	1.00	0.018
	April	0.86	0.85	0.88	0.000	0.91	0.90	0.92	0.000	0.96	0.95	0.97	0.000
	May	0.88	0.86	0.89	0.000	0.90	0.89	0.91	0.000	0.97	0.95	0.98	0.000
	June	0.95	0.93	0.96	0.000	0.87	0.86	0.88	0.000	0.85	0.83	0.87	0.000
	July	0.92	0.91	0.93	0.000	0.94	0.93	0.95	0.000	0.96	0.94	0.99	0.003
	August	0.97	0.96	0.99	0.000	1.03	1.01	1.04	0.000	1.03	1.01	1.06	0.020
	September	1.02	1.00	1.04	0.054	1.04	1.02	1.05	0.000	1.03	1.00	1.06	0.046
	October	1.02	1.00	1.04	0.105	1.07	1.06	1.09	0.000	1.13	1.10	1.16	0.000
	November	0.89	0.88	0.91	0.000	1.02	1.01	1.03	0.000	1.15	1.13	1.18	0.000
	December	0.90	0.89	0.91	0.000	1.01	1.00	1.02	0.032	1.16	1.14	1.19	0.000
2021	January	0.95	0.94	0.96	0.000	1.02	1.01	1.03	0.000	1.11	1.09	1.13	0.000
	February	0.77	0.76	0.78	0.000	0.91	0.90	0.92	0.000	1.02	1.00	1.03	0.010
	March	1.00	0.99	1.02	0.639	1.08	1.07	1.09	0.000	1.11	1.10	1.12	0.000
	April	0.94	0.92	0.95	0.000	1.01	1.00	1.03	0.055	1.06	1.05	1.08	0.000
	May	0.92	0.91	0.94	0.000	0.98	0.97	1.00	0.011	1.13	1.11	1.15	0.000
	June	0.99	0.98	1.00	0.162	1.06	1.04	1.07	0.000	1.09	1.07	1.12	0.000
	July	1.06	1.04	1.07	0.000	1.12	1.10	1.14	0.000	1.20	1.16	1.23	0.000
	August	1.01	0.99	1.03	0.450	1.14	1.13	1.16	0.000	1.22	1.18	1.26	0.000
	September	0.95	0.93	0.97	0.000	1.04	1.03	1.06	0.000	1.18	1.14	1.23	0.000
	October	0.97	0.94	0.99	0.002	1.11	1.09	1.12	0.000	1.22	1.18	1.26	0.000
	November	1.04	1.02	1.06	0.000	1.14	1.12	1.15	0.000	1.24	1.20	1.28	0.000
	December	0.99	0.98	1.01	0.258	1.09	1.08	1.10	0.000	1.13	1.10	1.16	0.000
**C. Amazon**													
2020	March	0.87	0.86	0.88	0.000	0.96	0.94	0.98	0.000	1.09	1.07	1.10	0.000
	April	0.78	0.76	0.79	0.000	0.89	0.87	0.91	0.000	0.88	0.87	0.90	0.000
	May	0.77	0.75	0.79	0.000	0.95	0.93	0.97	0.000	1.10	1.08	1.12	0.000
	June	0.75	0.73	0.78	0.000	0.82	0.80	0.84	0.000	0.88	0.85	0.90	0.000

	July	0.84	0.82	0.87	0.000	0.95	0.92	0.98	0.000	1.01	0.98	1.05	0.429
	August	0.92	0.89	0.95	0.000	0.96	0.93	0.99	0.020	0.95	0.92	0.99	0.007
	September	0.89	0.87	0.91	0.000	1.03	1.00	1.07	0.075	1.09	1.05	1.13	0.000
	October	1.00	0.98	1.02	0.911	1.05	1.02	1.09	0.005	1.07	1.03	1.11	0.000
	November	0.83	0.82	0.84	0.000	0.93	0.90	0.97	0.000	1.03	1.00	1.07	0.039
	December	0.89	0.88	0.90	0.000	0.92	0.89	0.94	0.000	1.06	1.03	1.09	0.000
2021	January	0.87	0.86	0.88	0.000	1.01	0.98	1.04	0.554	0.93	0.91	0.95	0.000
	February	0.79	0.78	0.80	0.000	0.86	0.84	0.89	0.000	1.12	1.10	1.15	0.000
	March	1.01	1.00	1.03	0.131	1.22	1.18	1.25	0.000	1.30	1.28	1.33	0.000
	April	1.15	1.12	1.18	0.000	1.20	1.16	1.24	0.000	1.35	1.32	1.39	0.000
	May	0.97	0.94	1.00	0.055	1.17	1.13	1.20	0.000	1.25	1.21	1.29	0.000
	June	1.04	1.00	1.07	0.051	1.12	1.08	1.16	0.000	1.21	1.17	1.26	0.000
	July	1.07	1.03	1.11	0.001	1.23	1.18	1.28	0.000	1.34	1.28	1.40	0.000
	August	1.17	1.13	1.21	0.000	1.24	1.19	1.30	0.000	1.24	1.18	1.30	0.000
	September	1.09	1.06	1.13	0.000	1.23	1.18	1.29	0.000	1.31	1.25	1.38	0.000
	October	1.06	1.03	1.08	0.000	1.14	1.09	1.20	0.000	1.29	1.23	1.36	0.000
	November	1.10	1.08	1.12	0.000	1.21	1.15	1.26	0.000	1.30	1.25	1.36	0.000
	December	1.09	1.07	1.11	0.000	1.14	1.09	1.19	0.000	1.20	1.15	1.24	0.000

Note: Values represent the change in the IRR for each month during pandemic period compared to pre-pandemic period. Peru is geographically divided in three natural regions (Coast (A), Highlands (B) and Amazon (C)). Abbreviations: IRR – incidence rate ratio, 95% CI – 95% confidence interval.

Table S3. Percent change in small vulnerable newborn by month from March 2020 to December 2021 in Brazil, compared to the expected numbers based on the preceding three years.

Timepoints (month year)	Observed	Expected	Observed 95% CI		Expected 95% CI		% change
**Preterm births**							
Mar 2020	29185	28264.65	27484.81	30885.19	26757.78	29771.53	3.26
Apr 2020	27584	27507.98	25883.81	29284.19	26001.11	29014.86	0.28
May 2020	27108	26853.98	25407.81	28808.19	25347.11	28360.86	0.95
Jun 2020	26130	25605.65	24429.81	27830.19	24098.78	27112.53	2.05
Jul 2020	25605	25062.98	23904.81	27305.19	23556.11	26569.86	2.16
Aug 2020	25540	25151.65	23839.81	27240.19	23644.78	26658.53	1.54
Sep 2020	22473	21698.32	20772.81	24173.19	20191.44	23205.19	3.57
Oct 2020	22753	21905.99	21052.81	24453.19	20399.11	23412.86	3.87
Nov 2020	20925	21124.32	19224.81	22625.19	19617.45	22631.19	-0.94
Dec 2020	22326	22767.65	20625.81	24026.19	21260.78	24274.53	-1.94
**2020 TOTAL**	**249629**	**245943.20**					**1.49**
Jan 2021	27801	28698.54	26104.87	29497.13	27200.11	30196.97	-3.13
Feb 2021	26822	27671.29	25125.87	28518.13	26172.86	29169.71	-3.69
Mar 2021	28266	27952.95	26569.87	29962.13	26353.84	29552.06	1.12
Apr 2021	27339	27196.28	25642.87	29035.13	25597.17	28795.40	0.52
May 2021	26633	26542.28	24936.87	28329.13	24943.17	28141.40	0.34
Jun 2021	25458	25293.95	23761.87	27154.13	23694.84	26893.06	0.65
Jul 2021	24958	24751.28	23261.87	26654.13	23152.17	26350.40	0.84
Aug 2021	24726	24839.95	23029.87	26422.13	23240.84	26439.06	-0.46
Sep 2021	22443	21386.62	20746.87	24139.13	19787.50	22985.73	4.94
Oct 2021	22488	21594.28	20791.87	24184.13	19995.17	23193.40	4.14
Nov 2021	21632	20812.62	19935.87	23328.13	19213.50	22411.73	3.94
Dec 2021	23726	22455.95	22029.87	25422.13	20856.84	24055.06	5.66
**2021 TOTAL**	**302292**	**299196**					**1.03**
**Low birthweight**							
Mar 2020	21449	21589.62	20131.80	22766.20	20328.29	22850.96	-0.65

Apr 2020	20067	20580.96	18749.80	21384.20	19319.62	21842.29	-2.50
May 2020	20304	21158.96	18986.80	21621.20	19897.62	22420.29	-4.04
Jun 2020	18838	19652.96	17520.80	20155.20	18391.62	20914.29	-4.14
Jul 2020	19266	19669.29	17948.80	20583.20	18407.95	20930.63	-2.05
Aug 2020	18639	19584.96	17321.80	19956.20	18323.62	20846.29	-4.83
Sep 2020	17712	18371.29	16394.80	19029.20	17109.95	19632.63	-3.59
Oct 2020	17961	18782.62	16643.80	19278.20	17521.29	20043.96	-4.37
Nov 2020	17204	18222.62	15886.80	18521.20	16961.29	19483.96	-5.59
Dec 2020	18656	19279.96	17338.80	19973.20	18018.62	20541.29	-3.24
**2020 TOTAL**	**190096**	**196893.20**					**-3.45**
Jan 2021	19567	20048.17	18262.25	20871.75	18792.02	21304.32	-2.40
Feb 2021	18319	19075.67	17014.25	19623.75	17819.52	20331.82	-3.97
Mar 2021	21433	21520.58	20128.25	22737.75	20229.28	22811.87	-0.41
Apr 2021	20464	20511.91	19159.25	21768.75	19220.61	21803.21	-0.23
May 2021	20738	21089.91	19433.25	22042.75	19798.61	22381.21	-1.67
Jun 2021	19537	19583.91	18232.25	20841.75	18292.61	20875.21	-0.24
Jul 2021	19534	19600.24	18229.25	20838.75	18308.95	20891.54	-0.34
Aug 2021	19063	19515.91	17758.25	20367.75	18224.61	20807.21	-2.32
Sep 2021	18605	18302.24	17300.25	19909.75	17010.95	19593.54	1.65
Oct 2021	18865	18713.58	17560.25	20169.75	17422.28	20004.87	0.81
Nov 2021	18286	18153.58	16981.25	19590.75	16862.28	19444.87	0.73
Dec 2021	19780	19210.91	18475.25	21084.75	17919.61	20502.21	2.96
**2021 TOTAL**	**234191**	**235326.6**					**-0.48**
**Small for gestational age**							
Mar 2020	17592	18486.38	15547.38	19636.62	16481.61	20491.15	-4.84
Apr 2020	16665	17581.38	14618.77	18711.23	15576.07	19586.7	-5.21
May 2020	17277	18777.38	15229.12	19324.88	16771.51	20783.25	-8.00
Jun 2020	15753	17256.05	13703.43	17802.57	15249.6	19262.49	-8.71
Jul 2020	16086	17400.38	14034.7	18137.3	15393.35	19407.41	-7.55
Aug 2020	15055	16798.05	13001.93	17108.07	14790.42	18805.67	-10.37
Sep 2020	14556	15940.05	12501.13	16610.88	13931.81	17948.29	-8.68

Oct 2020	15865	17775.38	13808.28	17921.72	15766.52	19784.25	-10.74
Nov 2020	15620	17221.05	13561.39	17678.61	15211.54	19230.55	-9.30
Dec 2020	17011	17930.38	14950.47	19071.53	15920.23	19940.54	-5.13
**2020 TOTAL**	**161480**	**175166.50**					**-7.81**
Jan 2021	14682	14948.49	12584.57	16779.43	12944.43	16952.55	-1.78
Feb 2021	13675	13864.74	11575.73	15774.27	11860,00	15869.48	-1.37
Mar 2021	17691	18171.84	15589.86	19792.14	16141.93	20201.74	-2.64
Apr 2021	16939	17266.84	14835.94	19042.06	15236.23	19297.44	-1.90
May 2021	17808	18462.84	15703.00	19913,00	16431.52	20494.15	-3.55
Jun 2021	16571	16941.50	14464.02	18677.98	14909.46	18973.54	-2.19
Jul 2021	16511	17085.84	14402,00	18620,00	15053.06	19118.61	-3.36
Aug 2021	16031	16483.50	13919.95	18142.05	14449.98	18517.03	-2.75
Sep 2021	15717	15625.50	13603.86	17830.14	13591.21	17659.79	0.59
Oct 2021	17295	17460.84	15179.74	19410.26	15425.77	19495.9	-0.95
Nov 2021	17129	16906.50	15011.59	19246.41	14870.65	18942.36	1.32
Dec 2021	18330	17615.84	16210.40	20449.60	15579.18	19652.49	4.05
**2021 TOTAL**	**198379**	**200834.30**					**-1.22**

Notes: The model was adjusted for each calendar month (seasonality), number of livebirths, and administrative level. Small Vulnerable Newborn was defined as preterm birth, low birthweight and small for gestational age babies. Abbreviations: 95% CI – 95% confidence interval.

Table S4. Incidence Rate Ratio (IRR) of preterm births, low birthweight and small for gestational age newborns during COVID-19 by region in Brazil, 2017-2021.

		Preterm births				Low birthweight				Small for gestational age			
**Year**	**Month**	**IRR**	**95% CI**		**p-value**	**IRR**	**95% CI**		**p-value**	**IRR**	**95% CI**		**p-value**
**A. Central-West**													
2020	March	1.02	1.00	1.03	0.022	1.05	1.03	1.07	0.000	1.04	0.99	1.09	0.089
	April	0.93	0.92	0.94	0.000	0.92	0.90	0.94	0.000	0.93	0.88	0.97	0.001
	May	0.94	0.92	0.95	0.000	0.94	0.92	0.96	0.000	0.92	0.87	0.96	0.000
	June	0.93	0.92	0.95	0.000	0.86	0.85	0.88	0.000	0.80	0.77	0.83	0.000
	July	0.96	0.94	0.99	0.004	0.93	0.91	0.94	0.000	0.85	0.82	0.88	0.000
	August	1.06	1.03	1.10	0.000	0.94	0.93	0.96	0.000	0.80	0.78	0.83	0.000
	September	0.95	0.91	0.99	0.012	0.94	0.92	0.95	0.000	0.84	0.82	0.86	0.000
	October	0.98	0.94	1.02	0.299	0.95	0.93	0.97	0.000	0.96	0.93	0.98	0.000
	November	0.81	0.78	0.85	0.000	0.82	0.81	0.84	0.000	0.91	0.88	0.94	0.000
	December	0.82	0.79	0.85	0.000	0.93	0.91	0.95	0.000	0.99	0.96	1.03	0.579
2021	January	1.01	0.98	1.04	0.670	0.93	0.91	0.95	0.000	0.85	0.81	0.89	0.000
	February	0.95	0.93	0.97	0.000	0.88	0.85	0.90	0.000	0.80	0.76	0.84	0.000
	March	0.99	0.97	1.01	0.366	1.03	1.00	1.06	0.050	1.04	0.98	1.11	0.195
	April	0.93	0.91	0.95	0.000	0.94	0.91	0.97	0.000	0.92	0.86	0.98	0.013
	May	0.92	0.90	0.94	0.000	0.96	0.93	0.99	0.005	0.98	0.92	1.05	0.575
	June	0.96	0.94	0.99	0.002	0.95	0.93	0.98	0.001	0.87	0.82	0.92	0.000
	July	0.97	0.94	1.01	0.097	0.92	0.90	0.95	0.000	0.86	0.81	0.90	0.000
	August	1.04	0.99	1.08	0.107	0.95	0.93	0.97	0.000	0.86	0.82	0.90	0.000
	September	0.99	0.95	1.05	0.822	0.99	0.96	1.01	0.278	0.91	0.88	0.95	0.000
	October	0.98	0.93	1.04	0.488	1.05	1.02	1.08	0.001	1.06	1.02	1.10	0.003
	November	0.88	0.83	0.92	0.000	0.93	0.90	0.95	0.000	1.02	0.98	1.07	0.357
	December	0.94	0.90	0.98	0.010	1.02	0.99	1.05	0.201	1.16	1.10	1.22	0.000

**B. North**													
2020	March	0.96	0.95	0.97	0.000	1.05	1.03	1.07	0.000	1.02	0.99	1.05	0.312
	April	0.92	0.91	0.94	0.000	0.91	0.89	0.93	0.000	0.92	0.89	0.95	0.000
	May	0.91	0.90	0.93	0.000	0.97	0.95	0.99	0.001	0.97	0.94	1.00	0.066
	June	0.93	0.92	0.95	0.000	0.91	0.89	0.93	0.000	0.91	0.88	0.94	0.000
	July	0.96	0.95	0.98	0.000	0.93	0.91	0.95	0.000	0.88	0.86	0.90	0.000
	August	0.97	0.96	0.99	0.000	0.89	0.88	0.91	0.000	0.81	0.80	0.83	0.000
	September	0.86	0.85	0.87	0.000	0.86	0.85	0.88	0.000	0.78	0.76	0.79	0.000
	October	0.96	0.94	0.97	0.000	0.94	0.92	0.96	0.000	0.90	0.89	0.92	0.000
	November	0.82	0.81	0.83	0.000	0.88	0.86	0.89	0.000	0.89	0.88	0.91	0.000
	December	0.81	0.80	0.82	0.000	0.92	0.90	0.94	0.000	1.02	1.00	1.05	0.055
2021	January	1.01	0.99	1.02	0.328	1.00	0.98	1.02	0.800	0.85	0.83	0.88	0.000
	February	0.93	0.91	0.94	0.000	0.92	0.90	0.94	0.000	0.83	0.80	0.85	0.000
	March	0.94	0.92	0.95	0.000	1.04	1.02	1.07	0.001	1.05	1.01	1.09	0.009
	April	0.90	0.89	0.92	0.000	1.00	0.97	1.02	0.832	1.04	1.00	1.08	0.063
	May	0.90	0.88	0.91	0.000	1.00	0.97	1.03	0.905	1.02	0.98	1.07	0.241
	June	0.91	0.89	0.93	0.000	0.92	0.89	0.95	0.000	0.95	0.92	0.99	0.006
	July	0.95	0.93	0.97	0.000	1.00	0.97	1.03	0.796	0.95	0.92	0.98	0.003
	August	0.98	0.97	1.00	0.094	0.92	0.89	0.95	0.000	0.89	0.86	0.91	0.000
	September	0.96	0.95	0.98	0.000	0.97	0.94	0.99	0.016	0.88	0.86	0.90	0.000
	October	1.01	1.00	1.03	0.093	1.03	1.00	1.06	0.046	1.01	0.98	1.03	0.565
	November	0.93	0.92	0.95	0.000	1.00	0.98	1.03	0.831	1.02	0.99	1.05	0.164
	December	0.94	0.93	0.96	0.000	1.06	1.03	1.09	0.000	1.16	1.13	1.20	0.000
**C. Northeast**													
2020	March	1.02	1.00	1.03	0.025	1.03	1.01	1.05	0.001	1.05	1.00	1.09	0.044
	April	0.98	0.96	1.00	0.020	0.95	0.93	0.97	0.000	0.96	0.91	1.01	0.092
	May	1.00	0.98	1.02	0.976	0.97	0.95	0.99	0.003	0.99	0.94	1.05	0.792
	June	1.01	0.99	1.04	0.203	0.94	0.92	0.96	0.000	0.89	0.85	0.94	0.000

	July	1.05	1.03	1.07	0.000	0.94	0.92	0.95	0.000	0.88	0.84	0.93	0.000
	August	1.15	1.13	1.17	0.000	0.97	0.95	0.98	0.000	0.83	0.79	0.87	0.000
	September	1.01	0.99	1.03	0.287	0.92	0.91	0.94	0.000	0.79	0.76	0.82	0.000
	October	1.01	1.00	1.03	0.082	0.92	0.90	0.93	0.000	0.90	0.87	0.94	0.000
	November	0.91	0.90	0.92	0.000	0.89	0.88	0.91	0.000	0.90	0.87	0.94	0.000
	December	0.87	0.86	0.89	0.000	0.91	0.90	0.93	0.000	1.01	0.96	1.05	0.814
	January	1.07	1.05	1.08	0.000	0.96	0.94	0.98	0.000	0.86	0.82	0.90	0.000
	February	0.97	0.96	0.99	0.001	0.87	0.85	0.89	0.000	0.80	0.75	0.84	0.000
	March	1.00	0.99	1.02	0.614	1.01	0.99	1.04	0.316	1.02	0.96	1.09	0.468
	April	1.01	0.99	1.03	0.291	0.99	0.97	1.02	0.493	1.02	0.95	1.09	0.591
	May	1.01	0.98	1.03	0.677	1.01	0.99	1.04	0.298	1.06	0.99	1.14	0.100
2021	June	1.00	0.98	1.03	0.752	0.94	0.92	0.97	0.000	0.96	0.89	1.03	0.297
	July	1.04	1.01	1.06	0.008	0.96	0.93	0.98	0.001	0.94	0.88	1.01	0.095
	August	1.11	1.09	1.14	0.000	0.98	0.95	1.00	0.095	0.89	0.83	0.95	0.000
	September	1.03	1.01	1.05	0.016	1.00	0.97	1.02	0.700	0.90	0.84	0.96	0.001
	October	1.03	1.01	1.05	0.003	0.99	0.97	1.02	0.677	1.01	0.96	1.07	0.661
	November	0.95	0.93	0.97	0.000	0.96	0.93	0.99	0.002	1.05	0.99	1.12	0.089
	December	0.98	0.96	0.99	0.006	1.00	0.97	1.03	0.914	1.14	1.07	1.21	0.000
**D. South**													
	March	0.98	0.97	0.99	0.000	0.97	0.97	0.97	0.000	0.98	0.96	1.00	0.069
	April	0.95	0.94	0.97	0.000	0.94	0.94	0.94	0.000	0.99	0.96	1.01	0.310
	May	0.99	0.97	1.00	0.020	0.95	0.94	0.95	0.000	0.98	0.95	1.01	0.261
	June	0.98	0.97	0.99	0.000	0.90	0.90	0.90	0.000	0.88	0.85	0.90	0.000
2020	July	1.01	1.00	1.02	0.054	0.97	0.97	0.97	0.000	0.95	0.93	0.98	0.000
	August	1.03	1.02	1.04	0.000	0.93	0.92	0.93	0.000	0.89	0.87	0.91	0.000
	September	0.99	0.98	1.00	0.021	0.93	0.92	0.93	0.000	0.89	0.87	0.90	0.000
	October	0.97	0.96	0.98	0.000	0.97	0.97	0.98	0.000	0.97	0.96	0.99	0.001
	November	0.88	0.87	0.88	0.000	0.90	0.90	0.91	0.000	0.97	0.95	0.98	0.000

	December	0.97	0.96	0.98	0.000	0.98	0.98	0.99	0.000	1.05	1.03	1.07	0.000
2021	January	1.06	1.06	1.07	0.000	1.00	0.99	1.00	0.288	0.96	0.94	0.98	0.000
	February	0.96	0.95	0.97	0.000	0.85	0.85	0.86	0.000	0.88	0.86	0.90	0.000
	March	0.98	0.97	1.00	0.010	1.01	1.01	1.02	0.000	1.08	1.05	1.12	0.000
	April	0.94	0.92	0.95	0.000	0.93	0.93	0.93	0.000	0.96	0.92	1.00	0.041
	May	0.95	0.93	0.96	0.000	0.94	0.93	0.94	0.000	0.95	0.91	0.99	0.021
	June	0.96	0.95	0.98	0.000	0.94	0.94	0.94	0.000	0.94	0.90	0.98	0.003
	July	0.98	0.97	1.00	0.013	0.95	0.95	0.96	0.000	0.96	0.92	0.99	0.021
	August	1.00	0.99	1.02	0.605	0.96	0.96	0.97	0.000	0.96	0.93	0.99	0.018
	September	0.96	0.95	0.97	0.000	0.97	0.96	0.98	0.000	0.90	0.87	0.92	0.000
	October	0.95	0.94	0.96	0.000	0.94	0.93	0.95	0.000	1.04	1.02	1.07	0.001
	November	0.92	0.91	0.93	0.000	0.95	0.94	0.95	0.000	1.06	1.03	1.08	0.000
	December	0.92	0.91	0.93	0.000	0.94	0.94	0.95	0.000	1.09	1.06	1.12	0.000
**E. Southeast**													
2020	March	1.01	1.00	1.01	0.091	1.00	0.99	1.02	0.708	1.01	0.97	1.05	0.594
	April	0.93	0.92	0.94	0.000	0.95	0.93	0.96	0.000	0.94	0.90	0.98	0.003
	May	0.94	0.94	0.95	0.000	0.95	0.93	0.96	0.000	0.94	0.90	0.98	0.005
	June	0.98	0.97	0.98	0.000	0.89	0.88	0.91	0.000	0.84	0.81	0.88	0.000
	July	1.03	1.02	1.04	0.000	0.96	0.95	0.98	0.000	0.87	0.84	0.91	0.000
	August	1.08	1.06	1.09	0.000	0.95	0.94	0.97	0.000	0.84	0.81	0.87	0.000
	September	0.99	0.98	1.01	0.556	0.92	0.91	0.94	0.000	0.85	0.82	0.87	0.000
	October	1.00	0.97	1.02	0.731	0.94	0.93	0.96	0.000	0.91	0.88	0.94	0.000
	November	0.91	0.89	0.93	0.000	0.92	0.90	0.94	0.000	0.95	0.92	0.98	0.001
	December	0.93	0.91	0.95	0.000	0.99	0.96	1.01	0.185	1.04	1.00	1.08	0.049
2021	January	1.05	1.03	1.07	0.000	0.98	0.96	1.01	0.146	0.91	0.87	0.95	0.000
	February	0.96	0.95	0.97	0.000	0.90	0.88	0.92	0.000	0.82	0.78	0.87	0.000
	March	0.97	0.96	0.98	0.000	1.01	0.98	1.03	0.489	1.04	0.98	1.10	0.185
	April	0.92	0.91	0.93	0.000	0.95	0.92	0.97	0.000	0.94	0.88	1.00	0.047

May	0.94	0.93	0.95	0.000	0.97	0.94	0.99	0.006	0.98	0.92	1.04	0.454
June	0.95	0.94	0.96	0.000	0.94	0.91	0.96	0.000	0.89	0.84	0.95	0.000
July	1.02	1.00	1.03	0.037	0.97	0.95	1.00	0.024	0.89	0.84	0.94	0.000
August	1.05	1.03	1.07	0.000	0.98	0.96	1.00	0.082	0.91	0.86	0.96	0.000
September	0.97	0.95	0.99	0.009	0.94	0.92	0.96	0.000	0.91	0.86	0.95	0.000
October	0.96	0.94	0.99	0.006	0.98	0.95	1.00	0.089	1.00	0.95	1.04	0.913
November	0.91	0.89	0.94	0.000	0.95	0.92	0.97	0.000	1.00	0.96	1.05	0.934
December	0.98	0.95	1.00	0.046	1.03	1.00	1.07	0.029	1.08	1.02	1.14	0.004

Note: Values represent the change in the IRR for each month during pandemic period compared to pre-pandemic period. Brazil is geographically divided in five regions (Central-West (A), North (B), Northeast (C), South (D) and Southeast (E)). Abbreviations: IRR – incidence rate ratio, 95% CI – 95% confidence interval.

Table S5. Percent change in small vulnerable newborn by month from March 2020 to December 2021 in Peru after adjustment for antenatal care at least four, compared to the expected numbers based on the preceding three years.

Timepoints (month year)	Observed	Expected	Observed 95% CI		Expected 95% CI		% change
**Preterm births**							
Mar 2020	2775	2683	2380.92	2880.72	2394.43	2971.04	3.44
Apr 2020	2296	2453	2084.20	2585.37	2161.67	2745.17	-6.42
May 2020	2354	2529	2157.19	2658.60	2237.23	2821.12	-6.93
Jun 2020	2364	2314	1989.01	2503.90	2010.55	2618.19	2.14
Jul 2020	2406	2476	2113.52	2648.52	2164.68	2787.49	-2.83
Aug 2020	2535	2645	2289.54	2801.70	2345.80	2944.72	-4.17
Sep 2020	2542	2554	2232.95	2741.92	2258.70	2849.36	-0.47
Oct 2020	2606	2794	2434.26	2940.59	2501.18	3086.03	-6.72
Nov 2020	2250	2626	2218.15	2725.57	2332.24	2920.25	-14.33
Dec 2020	2286	2959	2279.97	2987.86	2664.82	3254.10	-22.76
**2020 TOTAL**	24414	26034					-6.22
Jan 2021	2461	3055	2162.17	2759.83	2758.37	3351.12	-19.44
Feb 2021	2321	2833	2021.05	2620.95	2534.58	3131.72	-18.08
Mar 2021	2772	3086	2471.45	3072.55	2779.12	3393.49	-10.18
Apr 2021	2730	2851	2428.12	3031.88	2540.57	3161.75	-4.25
May 2021	2723	2916	2420.57	3025.43	2603.79	3228.21	-6.62
Jun 2021	2640	2791	2336.80	2943.20	2477.04	3104.90	-5.41
Jul 2021	2921	2873	2610.37	3231.63	2550.09	3196.65	1.66
Aug 2021	2989	3046	2683.45	3294.55	2727.12	3365.54	-1.88
Sep 2021	2721	2798	2415.12	3026.88	2477.70	3118.42	-2.75
Oct 2021	2668	2925	2360.61	2975.39	2601.59	3248.20	-8.78
Nov 2021	2761	2828	2452.88	3069.12	2502.62	3153.20	-2.37
Dec 2021	2894	3068	2585.26	3202.74	2740.32	3395.23	-5.66
**2021 TOTAL**	32601	35071					-7.04
**Low birthweight**							
Mar 2020	2517	2426	2213.39	2722.42	2269.59	2581.62	3.77

Apr 2020	2185	2236	1995.05	2507.95	2077.21	2394.84	-2.28
May 2020	2328	2295	2069.41	2585.66	2137.77	2451.30	1.46
Jun 2020	2116	2105	1872.49	2402.24	1938.62	2270.70	0.54
Jul 2020	2314	2233	2000.79	2547.08	2055.96	2409.17	3.65
Aug 2020	2339	2282	2042.80	2576.72	2124.34	2439.31	2.51
Sep 2020	2289	2233	1976.33	2511.14	2082.01	2383.20	2.53
Oct 2020	2341	2371	2083.60	2619.79	2226.36	2516.07	-1.27
Nov 2020	2128	2236	1929.26	2469.64	2092.18	2380.48	-4.84
Dec 2020	2125	2501	2119.27	2663.43	2359.35	2642.94	-15.04
**2020 TOTAL**	22682	22917					-1.02
Jan 2021	2267	2585	1963.00	2571.00	2444.98	2725.86	-12.32
Feb 2021	2191	2407	1884.55	2497.45	2265.40	2548.07	-8.96
Mar 2021	2611	2718	2302.50	2919.50	2566.36	2868.87	-3.92
Apr 2021	2520	2525	2208.89	2831.11	2371.50	2678.19	-0.19
May 2021	2570	2574	2256.81	2883.19	2421.98	2726.91	-0.17
Jun 2021	2506	2455	2190.57	2821.43	2303.15	2606.33	2.09
Jul 2021	2650	2526	2327.86	2972.14	2362.92	2688.85	4.91
Aug 2021	2647	2572	2326.53	2967.47	2419.98	2723.15	2.93
Sep 2021	2431	2399	2108.51	2753.49	2248.70	2549.88	1.32
Oct 2021	2435	2446	2109.67	2760.33	2293.93	2598.28	-0.45
Nov 2021	2442	2369	2114.39	2769.61	2217.42	2519.80	3.10
Dec 2021	2526	2559	2196.15	2855.85	2408.31	2710.10	-1.30
**2021 TOTAL**	29796	30134					-1.12
**Small for gestational age**							
Mar 2020	2051	2019	1866.92	2407.89	1872.42	2165.30	1.59
Apr 2020	1928	1898	1763.07	2310.21	1749.03	2047.17	1.58
May 2020	2078	1875	1771.54	2324.53	1727.40	2021.69	10.85
Jun 2020	1781	1757	1614.32	2181.83	1601.14	1912.86	1.37
Jul 2020	1972	1804	1669.54	2253.35	1638.09	1969.64	9.32
Aug 2020	2012	1767	1636.38	2213.41	1618.89	1914.54	13.88
Sep 2020	1928	1826	1618.56	2199.46	1684.37	1967.08	5.60

Oct 2020	1966	1832	1609.34	2194.56	1695.68	1967.62	7.33
Nov 2020	1905	1680	1482.24	2074.14	1544.89	1815.52	13.38
Dec 2020	1862	1851	1581.91	2180.17	1717.42	1983.62	0.62
**2020 TOTAL**	19483	18307					6.42
Jan 2021	1744	1945	1417.88	2070.12	1812.81	2076.47	-10.32
Feb 2021	1809	1833	1479.28	2138.72	1700.77	1966.10	-1.33
Mar 2021	2154	2171	1821.02	2486.98	2028.67	2312.63	-0.77
Apr 2021	2073	2048	1736.28	2409.72	1904.38	2192.26	1.20
May 2021	2143	2019	1802.97	2483.03	1875.46	2161.69	6.16
Jun 2021	2104	1949	1760.52	2447.48	1806.23	2090.81	7.98
Jul 2021	2197	1959	1846.54	2547.47	1806.24	2112.18	12.14
Aug 2021	2099	1917	1748.15	2449.85	1774.56	2059.14	9.50
Sep 2021	2020	1892	1665.85	2374.15	1751.09	2033.79	6.74
Oct 2021	2038	1835	1679.88	2396.12	1691.81	1977.50	11.08
Nov 2021	2015	1723	1653.36	2376.64	1581.15	1864.99	16.94
Dec 2021	1933	1843	1567.87	2298.13	1700.97	1984.26	4.91
**2021 TOTAL**	24329	23133					5.17

Notes: The model was adjusted for each calendar month (seasonality), number of livebirths, administrative level, and antenatal care at least four. Small Vulnerable Newborn was defined as preterm birth, low birthweight and small for gestational age babies. Abbreviations: 95% CI – 95% confidence interval.

Table S6. Percent change in small vulnerable newborn by month from March 2020 to December 2021 in Brazil after adjustment for antenatal care at least four, compared to the expected numbers based on the preceding three years.

Timepoints (month year)	Observed	Expected	Observed 95% CI		Expected 95% CI		% change
**Preterm births**							
Mar 2020	29185	29215	26991.75	31066.24	27079.25	31351.30	-0.10
Apr 2020	27584	28545	26058.74	30193.96	26390.03	30699.03	-3.37
May 2020	27108	26972	24723.13	28918.00	24796.21	29147.07	0.51
Jun 2020	26130	25939	23670.54	27935.88	23740.48	28137.40	0.74
Jul 2020	25605	25678	23317.13	27640.15	23463.03	27893.52	-0.29
Aug 2020	25540	25322	22994.36	27389.35	23080.24	27564.07	0.86
Sep 2020	22473	22853	20356.58	24805.82	20597.94	25108.93	-1.66
Oct 2020	22753	22514	20127.74	24656.36	20227.03	24800.09	1.06
Nov 2020	20925	21770	19080.23	23674.40	19460.75	24078.31	-3.88
Dec 2020	22326	23862	20979.55	25621.78	21542.97	26181.73	-6.44
**2020 TOTAL**	249629	252670					-1.20
Jan 2021	27801	29397	25288.16	30313.84	26985.24	31809.46	-5.43
Feb 2021	26822	28439	24274.14	29369.86	26001.38	30877.56	-5.69
Mar 2021	28266	28671	25698.26	30833.74	26188.89	31153.42	-1.41
Apr 2021	27339	27899	24737.76	29940.24	25393.33	30404.17	-2.01
May 2021	26633	26935	24002.89	29263.11	24408.88	29460.25	-1.12
Jun 2021	25458	25874	22789.14	28126.86	23320.09	28428.03	-1.61
Jul 2021	24958	25411	22259.96	27656.04	22838.24	27984.43	-1.78
Aug 2021	24726	25660	21995.89	27456.11	23064.30	28254.84	-3.64
Sep 2021	22443	22841	19680.36	25205.64	20223.09	25458.86	-1.74
Oct 2021	22488	22842	19688.42	25287.57	20196.02	25488.32	-1.55
Nov 2021	21632	22410	18800.77	24463.23	19741.45	25077.96	-3.47
Dec 2021	23726	24203	20867.31	26584.69	21518.96	26888.00	-1.97
**2021 TOTAL**	302292	310583					-2.67
**Low birthweight**							
Mar 2020	21449	21673	20970.70	21732.79	21203.83	22143.09	-1.04

Apr 2020	20067	20726	19920.41	20675.94	20259.12	21193.76	-3.18
May 2020	20304	20645	19894.65	20651.20	20176.05	21114.28	-1.65
Jun 2020	18838	19294	18524.29	19272.68	18828.14	19759.61	-2.36
Jul 2020	19266	19513	18803.00	19550.41	19047.64	19977.43	-1.26
Aug 2020	18639	19109	18330.78	19081.16	18642.58	19575.58	-2.46
Sep 2020	17712	18602	16745.27	18492.77	18137.27	19066.41	-4.78
Oct 2020	17961	18620	17802.80	18559.41	18151.99	19088.68	-3.54
Nov 2020	17204	18087	16213.59	17972.56	17617.97	18556.71	-4.88
Dec 2020	19002	19467	18627.00	19377.93	19001.21	19933.12	-2.39
**2020 TOTAL**	190442	195737					-2.71
Jan 2021	19567	20030	19035.66	20098.34	19542.55	20517.51	-2.31
Feb 2021	18319	19107	17787.77	18850.23	18619.39	19595.22	-4.13
Mar 2021	21433	21367	20898.46	21967.54	20845.11	21888.50	0.31
Apr 2021	20464	20347	19932.92	20995.08	19828.01	20865.68	0.58
May 2021	20738	20702	20205.13	21270.87	20181.10	21223.49	0.17
Jun 2021	19537	19331	19006.52	20067.48	18812.35	19849.77	1.07
Jul 2021	19534	19405	19003.89	20064.11	18886.56	19922.94	0.67
Aug 2021	19063	19435	18532.79	19593.21	18916.77	19953.03	-1.91
Sep 2021	18605	18677	18074.49	19135.51	18158.29	19194.97	-0.38
Oct 2021	18865	18940	18332.65	19397.35	18419.71	19459.95	-0.40
Nov 2021	18286	18630	17753.61	18818.39	18109.93	19150.81	-1.85
Dec 2021	19780	19796	19249.09	20310.91	19276.78	20314.50	-0.08
**2021 TOTAL**	234191	235766					-0.67
**Small for gestational age**							
Mar 2020	17592	18174	16520.70	18654.25	17532.44	18815.42	-3.20
Apr 2020	16665	17321	15641.02	17798.08	16682.25	17958.92	-3.78
May 2020	17277	17964	16224.01	18404.50	17323.36	18604.94	-3.83
Jun 2020	15753	16572	14794.30	17003.90	15936.29	17208.62	-4.94
Jul 2020	16086	16886	15114.53	17346.11	16251.30	17521.34	-4.74
Aug 2020	15055	16017	14169.28	16431.60	15379.37	16653.80	-6.00
Sep 2020	14556	15751	13878.96	16161.14	15115.96	16385.12	-7.58

Oct 2020	15865	17257	15293.68	17611.33	16616.96	17896.42	-8.06
Nov 2020	15620	16725	14821.86	17166.44	16083.85	17366.12	-6.61
Dec 2020	17011	17705	15916.50	18276.73	17068.08	18341.02	-3.92
**2020 TOTAL**	161480	170371					-5.22
Jan 2021	14682	14601	13376.93	15987.07	13935.06	15266.81	0.56
Feb 2021	13675	13559	12355.21	14994.79	12892.43	14225.36	0.86
Mar 2021	17691	17615	16365.28	19016.72	16902.34	18327.55	0.43
Apr 2021	16939	16700	15599.74	18278.26	15991.79	17409.18	1.43
May 2021	17808	17710	16457.54	19158.46	16998.08	18421.92	0.55
Jun 2021	16571	16302	15203.82	17938.18	15593.06	17010.12	1.65
Jul 2021	16511	16494	15132.63	17889.37	15786.17	17201.80	0.10
Aug 2021	16031	15988	14639.69	17422.31	15279.84	16695.33	0.27
Sep 2021	15717	15511	14312.46	17121.54	14803.16	16219.22	1.33
Oct 2021	17295	17222	15874.50	18715.50	16511.93	17932.85	0.42
Nov 2021	17129	16878	15695.82	18562.18	16167.11	17588.89	1.49
Dec 2021	18330	17678	16886.87	19773.13	16969.04	18386.52	3.69
**2021 TOTAL**	198379	196258					1.08

Notes: The model was adjusted for each calendar month (seasonality), number of livebirths, administrative level, and antenatal care at least four. Small Vulnerable Newborn was defined as preterm birth, low birthweight and small for gestational age babies. Abbreviations: 95% CI – 95% confidence interval.

Table S7. Incidence Rate Ratio (IRR) of preterm births, low birthweight and small for gestational age newborns during COVID-19 in Peru adjusting for antenatal care at least four visits, 2017-2021.

		Preterm birth				Low birthweight				Small for gestational age			
**Year**	**Month**	**IRR**	**95% CI**		**p-value**	**IRR**	**95% CI**		**p-value**	**IRR**	**95% CI**		**p-value**
2020	March	0.99	0.97	1.01	0.254	0.99	0.98	1.00	0.210	1.01	0.99	1.02	0.468
	April	0.82	0.81	0.84	0.000	0.86	0.84	0.87	0.000	0.93	0.92	0.94	0.000
	May	0.85	0.83	0.86	0.000	0.92	0.91	0.93	0.000	1.00	0.99	1.01	0.899
	June	0.88	0.86	0.89	0.000	0.85	0.84	0.86	0.000	0.88	0.87	0.89	0.000
	July	0.89	0.88	0.91	0.000	0.95	0.93	0.96	0.000	1.00	0.99	1.01	0.701
	August	0.94	0.93	0.95	0.000	0.97	0.96	0.98	0.000	1.05	1.04	1.06	0.000
	September	0.92	0.91	0.93	0.000	0.95	0.95	0.96	0.000	1.03	1.02	1.05	0.000
	October	0.92	0.91	0.93	0.000	0.97	0.96	0.97	0.000	1.07	1.05	1.08	0.000
	November	0.79	0.78	0.79	0.000	0.88	0.87	0.88	0.000	1.04	1.02	1.05	0.000
	December	0.78	0.77	0.78	0.000	0.85	0.85	0.86	0.000	1.00	0.98	1.01	0.514
2021	January	0.82	0.81	0.83	0.000	0.89	0.88	0.90	0.000	0.91	0.90	0.92	0.000
	February	0.77	0.76	0.78	0.000	0.84	0.84	0.85	0.000	0.91	0.90	0.92	0.000
	March	0.92	0.91	0.93	0.000	0.99	0.98	1.00	0.032	1.05	1.04	1.06	0.000
	April	0.91	0.89	0.92	0.000	0.95	0.94	0.96	0.000	1.00	0.99	1.01	0.379
	May	0.91	0.90	0.93	0.000	0.97	0.97	0.98	0.000	1.03	1.02	1.04	0.000
	June	0.90	0.89	0.91	0.000	0.96	0.96	0.97	0.000	1.03	1.02	1.04	0.000
	July	1.01	1.00	1.02	0.177	1.04	1.03	1.05	0.000	1.11	1.10	1.12	0.000
	August	1.03	1.02	1.03	0.000	1.06	1.05	1.06	0.000	1.09	1.08	1.11	0.000
	September	0.93	0.93	0.94	0.000	0.98	0.98	0.99	0.000	1.09	1.07	1.11	0.000
	October	0.91	0.90	0.92	0.000	0.99	0.98	1.00	0.023	1.12	1.10	1.14	0.000
	November	0.92	0.91	0.93	0.000	0.98	0.97	0.99	0.000	1.11	1.09	1.13	0.000
	December	0.95	0.95	0.96	0.000	1.00	0.99	1.01	0.949	1.05	1.03	1.06	0.000

Note: Values represent the change in the IRR for each month during pandemic period compared to pre-pandemic period. Abbreviations: IRR – incidence rate ratio, 95% CI – 95% confidence interval.

Table S8. Incidence Rate Ratio (IRR) of preterm births, low birthweight and small for gestational age newborns during COVID-19 in Brazil adjusting for antenatal care at least four visits, 2017-2021.

		Preterm birth				Low birthweight				Small for gestational age			
**Year**	**Month**	**IRR**	**95% CI**		**p-value**	**IRR**	**95% CI**		**p-value**	**IRR**	**95% CI**		**p-value**
2020	March	1.00	0.99	1.01	0.650	1.00	1.00	1.00	0.517	1.00	0.98	1.01	0.549
	April	0.95	0.94	0.96	0.000	0.96	0.96	0.97	0.000	0.99	0.97	1.00	0.114
	May	0.97	0.96	0.98	0.000	0.98	0.97	0.99	0.000	1.01	0.99	1.02	0.326
	June	1.00	0.98	1.01	0.860	0.97	0.96	0.98	0.000	0.99	0.97	1.01	0.399
	July	1.03	1.02	1.05	0.000	1.00	0.99	1.01	0.403	0.96	0.94	0.98	0.000
	August	1.09	1.07	1.11	0.000	1.00	0.99	1.01	0.415	0.92	0.90	0.94	0.000
	September	0.98	0.97	0.99	0.004	0.93	0.92	0.94	0.000	0.84	0.83	0.85	0.000
	October	1.00	0.98	1.01	0.778	0.96	0.96	0.97	0.000	0.96	0.94	0.98	0.000
	November	0.89	0.88	0.91	0.000	0.93	0.93	0.94	0.000	1.00	0.98	1.02	0.947
	December	0.90	0.89	0.91	0.000	0.97	0.97	0.97	0.000	1.06	1.04	1.07	0.000
2021	January	1.05	1.04	1.07	0.000	0.99	0.99	0.99	0.000	0.92	0.90	0.93	0.000
	February	0.98	0.96	0.99	0.004	0.95	0.95	0.95	0.000	0.93	0.91	0.95	0.000
	March	0.98	0.97	0.99	0.003	1.01	1.01	1.02	0.000	1.04	1.02	1.06	0.000
	April	0.96	0.95	0.97	0.000	1.00	0.99	1.01	0.764	1.05	1.03	1.07	0.000
	May	0.96	0.95	0.97	0.000	1.00	0.99	1.01	0.980	1.05	1.03	1.07	0.000
	June	0.98	0.97	1.00	0.045	1.01	0.99	1.02	0.256	1.05	1.02	1.08	0.000
	July	1.02	1.00	1.04	0.014	1.02	1.00	1.03	0.015	1.01	0.99	1.03	0.446
	August	1.06	1.05	1.08	0.000	1.01	0.99	1.02	0.331	0.97	0.94	0.99	0.002
	September	0.99	0.97	1.00	0.150	0.97	0.97	0.98	0.000	0.92	0.90	0.93	0.000
	October	0.99	0.98	1.01	0.356	1.00	0.99	1.01	0.820	1.03	1.02	1.05	0.000
	November	0.93	0.91	0.94	0.000	0.97	0.97	0.98	0.000	1.06	1.04	1.08	0.000
	December	0.96	0.95	0.98	0.000	1.02	1.01	1.02	0.000	1.12	1.11	1.14	0.000

Note: Values represent the change in the IRR for each month during pandemic period compared to pre-pandemic period. Abbreviations: IRR – incidence rate ratio, 95% CI – 95% confidence interval.

Appendix S1. Abstract in Spanish and Portuguese

Resumen (Abstract in Spanish)

Antecedentes: Examinamos el impacto de la COVID-19 en el número de recién nacidos pequeños y vulnerables (SVN) a nivel nacional y regional en Perú y Brasil.

Métodos: Utilizando los registros nacionales de nacimientos, examinamos los números mensuales de nacimientos prematuros (PT), de bajo peso al nacer (LBW) y de pequeños para la edad gestacional (SGA). Analizamos el impacto de la COVID-19 en los SVN utilizando dos modelos de series de tiempo interrumpidas. Estimamos los números esperados de SVN sin la pandemia mediante regresiones de efectos mixtos y calculamos los cambios porcentuales comparando estas estimaciones con los observados durante la pandemia. Las tasas de incidencia (IRR) fueron estimadas mediante regresión de Poisson.

Resultados: En Perú, los cambios porcentuales promedio en los nacimientos de PT, LBW y SGA fueron −17%, −11% y −3% en 2020, y −10%, −4% y +2% en 2021, respectivamente. La IRR de PT y LBW disminuyó durante la pandemia, mientras que la IRR de SGA aumentó entre agosto y noviembre de 2020 y de mayo a diciembre de 2021. La región natural de la Costa experimentó la mayor caída en la IRR de PT, LBW y SGA en 2020, seguida de un ligero aumento en 2021, mientras que las regiones naturales de los Andes y la Amazonía tuvieron un aumento en la IRR de LBW y SGA. En Brasil, los cambios porcentuales en los nacimientos de PT, LBW y SGA fueron +1%, −3% y −8% en 2020, y +1%, 0% y −1% en 2021, respectivamente. La mayoría de las IRR de PT, LBW y SGA disminuyó durante la pandemia, excepto en las regiones Nordeste y Sudeste, donde PT aumentó en 2020. Todas las regiones experimentaron disminuciones en LBW y SGA en 2020, siendo las regiones Centro-Oeste y Sur las que mostraron las mayores caídas de LBW y la región Centro-Oeste la mayor caída de SGA.

Conclusiones: No se observó un cambio significativo de los indicadores neonatales adversos en Perú y Brasil durante la pandemia de COVID-19. En Perú, los nacimientos de PT y LBW disminuyeron, mientras que SGA aumentó a partir de agosto de 2020. En Brasil, los nacimientos de PT aumentaron ligeramente, mientras que los nacimientos de LBW y SGA disminuyeron en 2020, manteniéndose estables en 2021.

Resumo (Abstract in Portuguese)

Antecedentes: Examinamos o impacto da COVID-19 no número de recém-nascidos pequenos e vulneráveis (SVN) em níveis nacional e regional no Peru e no Brasil.

Métodos: Com base nos registros nacionais de nascimento, examinamos os números mensais de recém-nascidos prematuros (PT), com baixo peso ao nascer (LBW) e pequenos para a idade gestacional (SGA). Analisamos o impacto da COVID-19 nos SVN utilizando dois modelos de séries temporais interrompidas. Estimamos os números esperados de SVN na ausência da pandemia utilizando regressões de efeitos mistos e calculamos as variações percentuais comparando essas estimativas com as observadas durante a pandemia. As razões de taxas de incidência (IRR) foram estimadas utilizando regressão de Poisson.

Resultados: No Peru, as variações percentuais médias nos nascimentos de PT, LBW e SGA foram de −17%, −11% e −3% em 2020, e −10%, −4% e +2% em 2021, respectivamente. A IRR de PT e LBW diminuiu durante toda a pandemia, enquanto a IRR de SGA aumentou entre agosto e novembro de 2020 e entre maio e dezembro de 2021. A região Costeira experimentou a maior queda na IRR de PT, LBW e SGA em 2020, seguida por um pequeno aumento em 2021, enquanto as regiões Andina e Amazônica apresentaram aumentos nas IRRs de LBW e SGA. No Brasil, as variações percentuais nos nascimentos de PT, LBW e SGA foram de +1%, −3% e −8% em 2020, e +1%, 0% e −1% em 2021, respectivamente. A maioria das IRRs de PT, LBW e SGA diminuiu durante a pandemia, exceto nas regiões Nordeste e Sudeste, onde PT aumentou em 2020. Todas as regiões experimentaram quedas em LBW e SGA em 2020, com as regiões Centro-Oeste e Sul apresentando as maiores quedas em LBW, e a região Centro-Oeste a maior queda em SGA.

Conclusões: Não foi observado um agravamento significativo dos desfechos neonatais no Peru e no Brasil durante a pandemia de COVID-19. No Peru, os nascimentos de PT e LBW diminuíram, enquanto os de SGA aumentaram a partir de agosto de 2020. No Brasil, os nascimentos de PT aumentaram ligeiramente, enquanto os de LBW e SGA diminuíram em 2020 e permaneceram estáveis em 2021.
